# An insect symbiotic virus promotes the transmission of a phytoarbovirus via inhibiting E3 ubiquitin ligase Sina

**DOI:** 10.1371/journal.ppat.1013178

**Published:** 2025-05-29

**Authors:** Hui Wang, Jieting Zhang, Runfa Liu, You Li, Yu Du, Taiyun Wei

**Affiliations:** State Key Laboratory of Agricultural and Forestry Biosecurity, Fujian Agriculture and Forestry University, Fuzhou, Fujian, China; University of California, Davis Genome Center, UNITED STATES OF AMERICA

## Abstract

Co-infection with symbiotic viruses and arboviruses with synergistic effects in insect vectors are common in nature, but the underlying mechanism remains elusive. Here, we identify a novel symbiotic virus, leafhopper *Recilia dorsalis* bunyavirus (RdBV), which enhances the transmission efficiency of cytorhabdovirus rice stripe mosaic virus (RSMV, a plant rhabdovirus) in field. RSMV infection activates the expression of *R. dorsalis* E3 ubiquitin ligase Seven in absentia (RdSina), while RdBV infection suppresses its expression. We show that RdSina directly targets and mediates the degradation of RSMV phosphoprotein (P), thereby attenuating the formation of P-induced viroplasm that are crucial for viral replication. RdSina interacts with nonstructural protein NSs2 of RdBV but does not mediate its ubiquitination. However, NSs2 competes with RSMV P for binding to RdSina, thus neutralizing RdSina’s ability in mediating P degradation. Furthermore, we find that the MYC transcription factor binds to the promoter sequences of RdSina, activating its transcription. However, NSs2 also directly binds to the same promoter sequences of RdSina and competitively suppresses MYC-activated RdSina transcription. Together, NSs2 obstructs the function of RdSina in mediating P degradation, ultimately promoting RSMV propagation in co-infected vectors. These findings elucidate how insect symbiotic viruses negatively regulate E3 ubiquitin ligases to benefit arbovirus transmission by co-infected insect vectors, which potentially is a common phenomenon in nature.

## Author summary

Many insects, such as mosquitoes, planthoppers, aphids and leafhoppers, are the hosts of symbiotic viruses and the vectors for transmitting arboviruses. Generally, symbiotic viruses can impact arbovirus transmission, but the mechanism remains largely unknown. Here, we demonstrate that symbiotic bunyavirus of leafhopper *Recilia dorsalis* (RdBV) promotes rice stripe mosaic virus (RSMV, a rhabdovirus) in field. RdBV-encoded NSs2 effectively suppresses the transcription of E3 ubiquitin ligase Seven in absentia (Sina). Furthermore, NSs2 competitively prevents Sina-mediated ubiquitinated degradation of RSMV-encoded phosphoprotein (P), ultimately promoting RSMV propagation and transmission. We believe that such a model of direct synergistic relationship between insect symbiotic viruses and arboviruses is a common phenomenon in nature.

## Introduction

Insects are the hosts of diverse symbiotic microorganisms, including viruses, bacteria, and fungi. Typically, insect symbiotic microorganisms reside within their insect hosts and are vertically transmitted from parents to offspring [1–3]. Insects also serve as important vectors for a wide range of arthropod-borne pathogenic viruses (arboviruses) and more than 70% of plant viruses and 17% of animal viruses are transmitted by specific arthropod vectors that include planthoppers, leafhoppers, aphids, whiteflies, and mosquitoes [4,5]. Co-infections with symbiotic microorganisms and arboviruses are common in nature [6,7]. Symbiotic viruses can suppress the infection of arboviruses in insect vectors [8,9]. For instance, cell-fusing agent virus negatively regulates the infection of both dengue and Zika viruses in mosquitoes, and palm creek virus suppresses the infection of West Nile virus in mosquitoes [8–11]. However, many symbiotic viruses are believed to benefit insect host fitness, potentially influencing the insect host’s ability to acquire and transmit arboviruses [1,12]. For example, Humaita Tubiacanga virus and Phasi Charoen-like virus promote dengue virus infection in mosquitoes [12]. Similarly, Himetobi P virus facilitates rice stripe virus infection in planthoppers [13]. Currently, the exact mechanisms by which insect symbiotic viruses and arboviruses establish direct synergistic interactions in nature are still poorly understood.

Leafhoppers (Cicadellidae), a large insect family containing approximately 22,000 described species, are cosmopolitan [14,15]. They feed on various crop plants and fruit trees and cause direct damage to agriculture production [15,16]. Around 200 leafhopper species can transmit pathogenic microbes, and thus the co-infections of symbiotic and pathogenic microbes are common [16,17]. During the past decade, we have used rice leafhopper *Recilia dorsalis* to investigate the complex interactions among arboviruses, symbiotic bacteria, and symbiotic viruses. Rice gall dwarf virus (RGDV), a plant reovirus, and a symbiotic virus of the *Virgaviridae* family cooperatively hijack insect sperm-specific proteins for paternal transmission via the direct interaction of their capsid proteins in *R. dorsalis* [1]. Two obligate symbiotic bacteria *Sulcia* and *Nasuia* in *R. dorsalis* harbor rice dwarf virus particles via the interaction of viral capsid protein and bacterial outer membrane proteins, facilitating transovarial viral transmission and thus forming a virus-bacterium synergistic interaction [18,19]. Moreover, RGDV enhances the transmission of rice stripe mosaic virus (RSMV, a plant rhabdovirus) by co-infected *R. dorsalis* [20]. RSMV directly utilizes RGDV-induced autophagosomes as sites for the assembly of enveloped virions in co-infected *R. dorsalis* via the direct interaction of RSMV-encoded nucleoprotein (N) with RGDV-encoded autophagy-induced protein [21]. Thus, RGDV promotes the transmission efficiency of RSMV [21]. It is intriguing for identification of leafhopper symbiotic viruses that enhance arbovirus transmission by *R.* dorsalis in their natural habitats.

RSMV, first identified in Luoding city, Guangdong Province, China since 2015, has spread to Hainan, Guangxi, Jiangxi, Hunan, and Yunnan provinces, resulting in considerable crop losses [22,23]. RSMV is mainly transmitted by *R. dorsalis* in a persistent-propagative manner [24]. The single-stranded negative genome RNA of RSMV encodes seven proteins in the order 3′-N-P-P3-M-G-P6-L-5′: N, phosphoprotein (P), nonstructural protein P3, matrix protein (M), glycoprotein (G), nonstructural protein P6, and large polymerase protein (L) [21]. Rhabdovirus transcription and replication follow a conserved mechanism across different viruses in the family [25]. After entering the host cell and releasing the nucleocapsid core (NC: RNA-N-P-L), the viral polymerase complex initiates transcription. Replication is thought to begin once the N protein reaches a sufficient concentration, triggering a switch from transcription to replication [26]. Rhabdoviruses induce the formation of viroplasms primarily composed of P proteins as the sites for viral replication and non-enveloped virion assembly [26,27]. The ribonucleoprotein (RNP) cores of RSMV are constructed inside the viroplasm, whereas the bacilliform non-enveloped virions are assembled at the viroplasm periphery [28,29]. Enveloped virions of RSMV are formed in the endoplasmic reticulum (ER) [28,29]. The cytorhabdovirus barley yellow striate mosaic virus P forms viroplasm-like granules with phase separation properties [27,30]. This highlights the essential role of rhabdovirus P proteins in viroplasm formation to support viral replication and virion assembly.

In this study, we demonstrated that E3 ubiquitin ligase Seven in absentia of *R. dorsalis* (RdSina) directly targeted and mediated the ubiquitinated degradation of RSMV P through the 26S proteasome pathway, thereby attenuating the formation of RSMV P-induced viroplasm. We identified a symbiotic bunyavirus, which benefits the reproduction of *R. dorsalis* and enhances the transmission efficiency of RSMV by co-infected *R. dorsalis* in the field. Symbiotic bunyavirus-encoded one nonstructural protein effectively suppressed the transcription of RdSina and competed with RSMV P for binding to RdSina, finally inhibiting RdSina-mediated degradation of RSMV P and benefiting RSMV propagation. This study reveals the mechanism of direct synergistic relationship between insect symbiotic viruses and arboviruses.

## Results

### Identification and characterization of a novel bunyavirus in *R. dorsalis*

**Fig 1:**
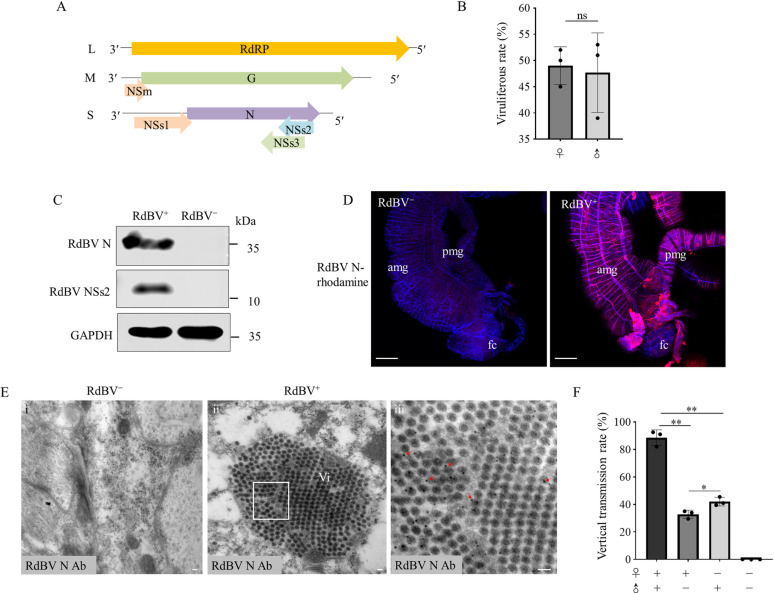
Characterization of a symbiotic bunya-like virus in *R. dorsalis.* (A) Genome organization of RdBV. Predicted functions of encoded proteins are indicated. (B) Viruliferous rates of male and female adults by RT-PCR assay for testing the transcription of RdBV N. Data are presented as means (± SD) of three independent biological replicates and each replicate contains 100 insects (two-tailed t test). ns, not significant. (C) Accumulation of RdBV N and NSs2 in 30 RdBV-positive and negative *R. dorsalis* individuals, as determined by western blot assays using RdBV N, NSs2 or GAPDH antibodies. Data represent three independent biological replicates. (D) Immunofluorescence microscopy showing the distribution of RdBV N in the intestines of RdBV-positive *R. dorsalis*. The dissected intestines of RdBV-positive or negative *R. dorsalis* were immunostained with RdBV N-rhodamine (red) and actin dye phalloidin-Alexa Fluor 647 (blue). Bars, 100 μm. (E) Immunoelectron microscopy showing the localization of RdBV N on spherical viral particles in the midgut epithelium of RdBV-positive *R. dorsalis*. The dissected intestines from RdBV-negative (i) and positive (ii) *R. dorsalis* individuals were immunolabeled with RdBV N antibody as the primary antibody, followed by treatment with 15-nm gold particle-conjugated IgG as the secondary antibody. Red arrows indicate gold particles. Panel iii is an enlarged image of the boxed area in panel ii. Bars, 100 nm. (F) Vertical transmission rates of RdBV by RdBV-positive and negative female and male *R. dorsalis* via mating. The offspring were tested for RdBV by RT-PCR assay. Data are presented as means (± SD) of three independent biological replicates and each replicate contains 50 offspring from each mating combination (two-tailed t test). *, *p* < 0.05; **, *p* < 0.01.

We identified a previously undescribed *Recilia dorsalis* bunyavirus (RdBV) belonging to order *Bunyavirales* from the field-caught *R. dorsalis* population in Guangdong Province, Southern China, in 2020 through analysis of RNA sequencing data. The RdBV genome comprised three segments of single-stranded, negative-sense or ambisense RNAs (GenBank accession No. PP666777, PP666778 and PP666779). The L segment was 6,636 nucleotides (nt) long and encoded RNA-dependent RNA polymerase (RdRp); the M segment was 4,372 nt long and encoded glycoprotein protein (G) and nonstructural protein NSm; the S segment was 1,461 nt long and encoded nucleocapsid (N) and three nonstructural proteins (NSs1-3) (Fig 1A). All the segments contained the conserved terminal sequences (5′- TAGCAGCACGCTG…CAGCATGCTGCT-3′) and the specific panhandle structures formed by inverted terminal repeats in the untranslated regions (UTR) (S1A, B Fig). Phylogenetic analysis revealed that RdBV was closely related to *Scaphoideus titanus* bunya-like virus 1 (StBV1), both belonging to genus *Cicadellivirus* in the family *Phasmaviridae* (S1C Fig). RdBV was detected at a prevalence of 45–50% in lab-reared male and female *R. dorsalis* population (Fig 1B). We prepared polyclonal antibody against RdBV N and NSs2, which specifically detected RdBV-positive *R. dorsalis* by western blot assay (Fig 1C). Immunofluorescence microscopy assay showed that RdBV N was distributed in the midguts, salivary glands, ovaries and testes of virus-positive *R. dorsalis* population (Figs 1D and S2). Immunoelectron microscopy confirmed that RdBV N antibody specifically reacted with the icosahedral viral particles (about 80 nm in diameter) in the cytoplasm of virus-infected midgut epithelium (Fig 1E).

To explore whether RdBV can be vertically transmitted, the offspring of four mating pairings, including infected females × infected males; infected females × uninfected males; uninfected females × infected males; and uninfected female × uninfected males were collected and tested. The mean percentages of 32.8%, 42%, and 88.5% was observed for RdBV from the offspring of infected females mated with uninfected males, uninfected females mated with infected males, and infected females mated with infected males, respectively (Fig 1F). Thus, paternal transmission of RdBV is more efficient than maternal transmission.

We then determined whether the efficient vertical RdBV transmission affected the fitness of *R. dorsalis* or their offspring. It was found that RdBV-positive males and females exhibited no significant differences in adult longevity compared with RdBV-negative controls (S3A Fig). To investigate the effect of RdBV infection on the fitness of *R. dorsalis* population, the eggs produced by two mating pairings, including one RdBV-positive virgin female × one RdBV-positive male and one RdBV-negative virgin female × one RdBV-negative male, were collected and analyzed. It was found that RdBV-positive parents laid about 16.9% more eggs compared to RdBV-negative parents, suggesting that RdBV infection benefits the reproduction of *R. dorsalis* (S3B Fig). The mean development duration for RdBV-positive eggs was no significant difference compared with RdBV-negative controls (S3C Fig).

### Co-infection of RdBV and RSMV promotes RSMV infection in *R. dorsalis*

**Fig 2:**
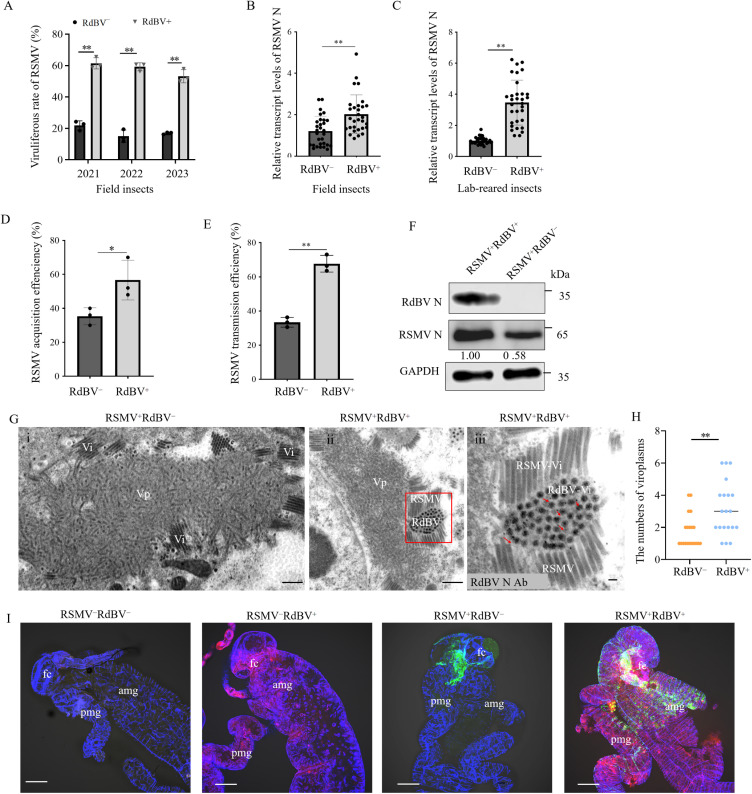
Co-infection of RdBV and RSMV promotes RSMV infection in *R. dorsalis.* (A) Viruliferous rates for *R. dorsalis* populations carrying RdBV or RSMV collected in Luoding field in Guangdong province, China for three consecutive years. Data represent the mean viruliferous rates of 100 insects tested by RT-qPCR assay from three biological replicates. (B and C) RT-qPCR assays showing the transcript levels of RSMV N in RdBV-positive and negative *R. dorsalis* populations from the diseased field (B) and the lab-reared (C) samples. (D) RSMV acquisition efficiencies by RdBV-positive and negative *R. dorsalis* populations, as determined by RT-qPCR assays for the presence of RSMV N (n = 200). (E) RSMV transmission efficiencies by RdBV-positive and negative *R. dorsalis* populations, as determined by RT-qPCR assays for the presence of RSMV N (n = 30). (F) Western blot assay showing the accumulation levels of RSMV N in 30 RdBV-positive or negative *R. dorsalis* individuals. (G) Immunoelectron microscopy showing the localization of RdBV N in RdBV and RSMV co-positive leafhopper midgut epithelial cells. The dissected intestines from single RSMV-positive (i) or RdBV and RSMV co-positive (ii) leafhoppers were immunolabeled with RdBV N antibody as the primary antibody, followed by treatment with 15-nm gold particle-conjugated IgG as the secondary antibody. Red arrows indicate gold particles. Panel iii is the enlarged image of the boxed area in panel ii. Bars, 500 μm (i and ii) and 100 nm (iii). (H) The mean number of viroplasms per midgut epithelial cell of single RSMV-positive or RdBV and RSMV co-positive leafhoppers is shown in Gi and Gii. (n = 20) (I) Immunofluorescence microscopy showing the distribution of RdBV N and RSMV N in RdBV or RSMV infected leafhopper intestines. The intestines of RdBV or RSMV infected leafhopper intestines were immunostained with RdBV N-rhodamine (red), RSMV N-FITC (green), or actin dye phalloidin-Alexa Fluor 647 (blue). Bars, 100 μm. Data in B-E are presented as means (± SD) of three independent biological replicates and each replicate contains 30 insects (two-tailed t test). *, *p* < 0.05; **, *p* < 0.01; ns, not significant. The proteins in F were detected by western blot assay using indicated antibodies. The relative intensities of the bands for these proteins are shown below using Image J. The data of western blot represent three biological replicates. Vp, viroplasm; Vi, virons; fc, filter chamber; amg, anterior midgut; pmg, posterior midgut.

As the common host of RSMV, *R. dorsalis* may become naturally infected with both RSMV and RdBV. We surveyed *R. dorsalis* populations collected during rice stripe mosaic disease outbreak in Luoding fields in Guangdong province, China over three consecutive years. The RdBV-positive field *R. dorsalis* population showed a higher viruliferous rates of RSMV than the RdBV-negative population (Fig 2A). RT-qPCR assay showed that the transcript levels of RSMV N in the RdBV-positive field-caught *R. dorsalis* population were significantly higher than that in the RdBV-negative field-caught population (Fig 2B). Thus, the vector competence of the field *R. dorsalis* population for RSMV is enhanced by RdBV.

Similarly, the RdBV-positive lab-reared *R. dorsalis* population also showed a higher competence for RSMV transmission than the RdBV-negative control (Fig 2C, 2F). Furthermore, the higher efficiencies of RSMV acquisition were observed in the RdBV -positive lab-reared *R. dorsalis* population (Fig 2D). To compare viral transmission efficiency, the rice plants were fed on by RdBV and RSMV co-positive or RSMV positive *R. dorsalis* for two days and then kept for testing viral titers. Western blot assays revealed a higher accumulation of viral proteins in rice plants fed on by RdBV and RSMV co-positive *R. dorsalis* (S4A Fig), thus led to a severer phenotype compared with the RSMV positive *R. dorsalis* (S4B Fig). Transmission efficiency by RdBV-positive lab-reared *R. dorsalis* population was 67.78% (average of 20/30, 22/30, and 19/30 plants), compared with 33.33% (average of 11/30, 9/30, and 10/30 plants) by RdBV-negative *R. dorsalis* population (Fig 2E). The presence of RSMV also increased RdBV titers (S5A, B Fig).

Electron microscopy showed that, in the RSMV singly infected midgut epithelial cells of *R. dorsalis*, the bacilliform non-enveloped particles of RSMV were assembled at the periphery of the viroplasm (Fig 2G). In the RdBV and RSMV co-positive midgut epithelial cells, the spherical particles of RdBV and the bacilliform non-enveloped particles of RSMV were accumulated together at the periphery of the viroplasm (Fig 2G). The average number of 1.75 RSMV viroplasms per midgut epithelial cell was calculated from at least 20 RdBV-positive cells observed (Fig 2H). However, the average number of 3.15 RSMV viroplasms per midgut epithelial cell was calculated from at least 20 RdBV-negative cells observed (Fig 2H). Thus, RdBV potentially promotes the formation of P-induced viroplasm. Immunofluorescence microscopy showed the co-infection of RSMV and RdBV in the same midgut epithelial cells (Fig 2I). Potentially, the co-infection of RSMV and RdBV in the same cells leads to RdBV-mediated increase of RSMV propagation.

### RdSina interacts with RSMV P and RdBV NSs2

**Fig 3:**
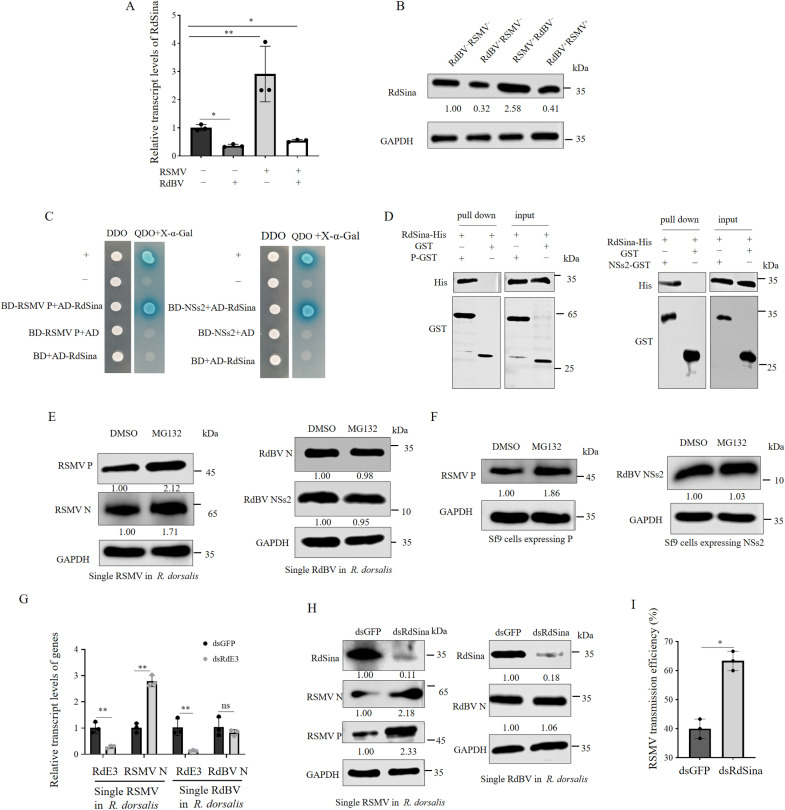
RdSina interacts with RdBV NSs2 or RSMV P. (A and B) RT-qPCR and western blot assays showing the relative transcript and protein levels of RdSina in RdBV^−^RSMV^−^, RdBV^+^RSMV^−^, RdBV^−^RSMV^+^, or RdBV^+^RSMV^+^ *R. dorsalis* individuals. (C) Y2H assays showing the interactions of RdSina with RdBV NSs2 or RSMV P. Yeast colonies are blue as shown in the β-galactosidase assay. (D) GST pull-down assays showing the interactions of RdSina with RdBV NSs2 or RSMV P. (E) Effects of the treatment with 10 μM MG132 or an equal volume of DMSO on the accumulation of RSMV- or RdBV-encoded proteins in single RSMV- or RdBV-positive *R. dorsalis* individuals, as determined by western blot assays. (F) Effects of the treatment with 10 μM MG132 or an equal volume of DMSO on the accumulation of RSMV P or RdBV NSs2 in Sf9 cells expressing P or NSs2, as determined by western blot assays. (G) RT-qPCR assay showing the relative transcript levels of RdSina, RSMV N, and RdBV N in dsGFP or dsRdSina-treated *R. dorsalis* individuals. (H) Western blot assay showing the accumulation of RSMV- or RdBV-encoded proteins in dsGFP or dsRdSina-treated *R. dorsalis* individuals. (I) Efficiencies of RSMV transmission to rice seedlings by dsRdSina- or dsGFP-treated leafhoppers with had been infected with RSMV, as calculated by the percentage of RT-PCR-positive plants out of the total number of tested plants. Data in A, G and I are presented as means (± SD) of three independent biological replicates and each replicate contains 30 insects (two-tailed t test). *, *p* < 0.05; **, *p* < 0.01; ns, not significant. The proteins in B, E, F and H were detected by western blot assay using indicated antibodies. The relative intensities of the bands for these proteins are shown below using Image J. The data of western blot represent three biological replicates.

We next investigated how RdBV improved RSMV propagation in co-infected insects. Transcriptome sequencing of RdBV-positive and negative *R. dorsalis* individuals showed that the presence of RdBV up-regulated the expression of the genes involved in the oxytocin and adrenergic signaling in cardiomyocytes pathways, but down-regulated the expression of the genes related to the mitogen-activated protein kinases (MAPK) signaling and ubiquitin-mediated proteolysis pathways (S6A, B Fig). Of these, an E3 ubiquitin ligase Sina (RdSina), a member of ubiquitin-mediated proteolysis pathways, was significantly down-regulated in the RdBV-positive population (S6A, B Fig). The full-length ORF of RdSina contained 807 nt and encoded 268 amino acid residues with a predicted molecular weight of 29.5 kDa, which had a canonical RING-Ubox type (C3HC4) domain at its N-terminus and a conserved Sina domain near the RING domain (S6C, D Fig). RdSina expression level was significantly down-regulated in the single RdBV-positive population but was significantly up-regulated in the single RSMV-positive population (Fig 3A, 3B). However, RSMV-mediated increase of RdSina could be restored by co-infection with RdBV and RSMV (Fig 3A, 3B). Thus, RdBV effectively suppresses the RSMV-mediated increase of RdSina.

Subsequently, RdSina was used as a bait protein to screen for interacting proteins encoded by RSMV, including N, P, M, G, P3 and P6 with yeast two-hybrid (Y2H) assay. It was found that RdSina specifically interacted with RSMV P (P), which has an estimated molecular weight of 42 kDa (Figs 3C and S7A). Meanwhile, Y2H assay also showed that RdSina interacted with NSs2 of RdBV, which has an estimated molecular weight of 10.2 kDa (NSs2), but not with other viral proteins encoded by RdBV (Figs 3C and S7B). Viral proteins encoded by RdBV and RSMV did not interact with each other (S7C Fig). GST pull-down assay confirmed that RdSina interacted with NSs2 and P (Fig 3D). Y2H assay further showed that both RdBV NSs2 and RSMV P interacted with the C-terminal region of RdSina, rather than with the RING domain-containing N-terminal region (S8A, B Fig).

The ubiquitination-proteasome system is one of the major protein degradation pathways, and the ubiquitination of protein is completed by a cascade reaction catalysed by the specific ubiquitin activating enzyme E1, ubiquitin binding enzyme E2 and ubiquitin ligase E3, where the substrate specificity is mainly determined by ubiquitin ligase [31,32]. We next investigated whether RdSina could mediate the ubiquitinated degradation of P or NSs2 via the 26S proteasome system. The proteasome inhibitor, MG132, was microinjected into single RSMV- or RdBV-positive *R. dorsalis*. Western blot assay showed that the protein accumulation levels of RSMV P and N were higher in the MG132 treated group compared to the DMSO control group, but that RdBV NSs2 and N were not significantly affected (Fig 3E). Electron microscopy showed there was a significant increase in the average number of RSMV viroplasms per midgut epithelial cell in the MG132 treated group compared to the DMSO control group (S9A, B Fig). Similar observations were made in Sf9 cells singly expressed with P or NSs2 (Fig 3F). Knockdown of RdSina expression via the microinjection of *in-vitro* synthesized dsRNAs targeting RdSina (dsRdSina) into single RSMV or RdBV positive *R. dorsalis* increased RSMV P and N accumulation levels, but had no significant impact on RdBV N accumulation levels (Fig 3G, 3H). In transmission efficiency experiments, leafhoppers were microinjected with dsRdSina and dsGFP to further investigate the function of RdSina in RSMV transmission by leafhoppers. The transmission efficiency by insects injected with dsRdSina was 63.33% (average of 20/30, 19/30, and 18/30 plants), compared with 40% (average of 12/30, 11/30, and 13/30 plants) after dsGFP injection (Fig 3I). The knockdown of RdSina expression also promoted RSMV accumulation level in the rice, thus leading to a severer phenotype compared with the dsGFP treatment *R. dorsalis* (S10A, B Fig). Potentially, RdSina could mediate the ubiquitinated degradation of P but not NSs2 in *R. dorsalis*, thus serving as a RSMV restriction factor and inhibiting the transmission of RSMV. However, RdBV potentially neutralizes this function of RdSina, and thus RdBV enhances the vector competence of co-infected *R. dorsalis* for RSMV.

### RdSina mediates RSMV P ubiquitinated degradation and negatively regulates RSMV propagation in leafhoppers

**Fig 4:**
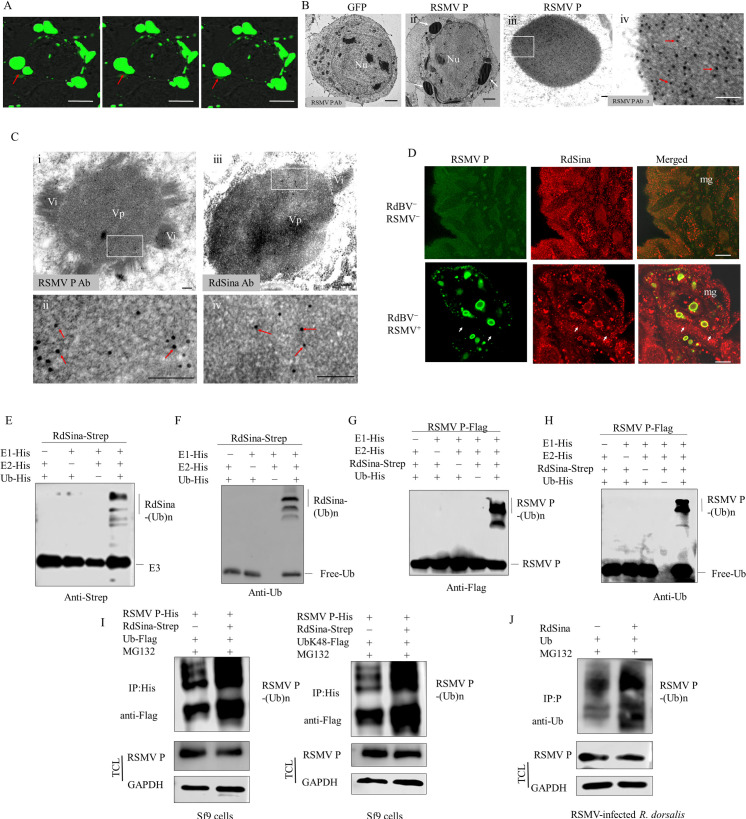
RdSina functions as an E3 ubiquitin ligase and mediates RSMV P ubiquitination. (A) Confocal microscopy showing the mobile punctate structures of RSMV P-GFP (arrows) in Sf9 cells expressing P-GFP. Bars, 10 μm. (B) Immunoelectron microscopy showing the appearance of RSMV P-formed granular inclusion bodies (white arrows) in Sf9 cells expressing P. Sf9 cells expressing GFP (i) or P (ii) were immunolabeled with P antibody, followed by treatment with 15-nm gold particle-conjugated IgG. Red arrows indicate gold particles. Panel iv is the enlarged image of the boxed area in panel iii. Bars, 2 μm (i and ii) and 100 nm (iii and iv). (C) Immunoelectron microscopy showing the localizations of RSMV P and RdSina in the viroplasm matrix in RSMV-infected leafhopper midgut epithelium. The dissected intestines from single RSMV-positive leafhoppers were immunolabeled with P (i and ii) or RdSina (iii and iv) antibody, followed by treatment with 15-nm gold particle-conjugated IgG. Red arrows indicate gold particles. Panels ii and iv are the enlarged images of the boxed areas in panels i and iii, respectively. Bars, 100 nm. (D) Immunofluorescence microscopy showing the co-localization of RdSina and RSMV P in single RSMV-positive leafhopper midgut epithelium. The dissected intestines from RSMV-positive or negative leafhoppers were immunostained with RSMV P-FITC (green) and RdSina-rhodamine (red). Arrows indicate the co-localization of P and RdSina. Bars, 100 μm. (E and F) The E3 ubiquitin ligase activity of RdSina. RdSina purified proteins were assayed for E3 ubiquitin ligase activity in the presence of E1, E2 and ubiquitin. Strep antibody was used to detect Strep-RdSina (E) and ubiquitin antibody was used to detect His-ubiquitin (F). (G and H) RdSina-mediated ubiquitination of RSMV P. Flag-P was used as a substrate for the assay. Flag antibody was used in the immunoblotting assay to detect Flag-P (G), and ubiquitin antibody was used to detect His-ubiquitin (H). (I) IP assay showing the ubiquitination modification of RSMV P by RdSina in Sf9 cells by co-expressing RSMV P-His, RdSina-Strep, and either ubiquitin-Flag or ubiquitin-K48-Flag in the presence of MG132. The P-His was immunoprecipitated by His antibody. The immunoprecipitated proteins were then detected using Flag antibody, while the TCL was detected by His or GAPDH antibody. (J) IP assay showing the ubiquitination modification of RSMV P by RdSina in RSMV-infected *R. dorsalis* by co-microinjecting RdSina and ubiquitin in the presence of MG132. RSMV P was immunoprecipitated by P antibody. The immunoprecipitated proteins were then detected using ubiquitin antibody, while the TCL was detected by GAPDH or P antibody. Nu, nucleus; Vp, viroplasm; Vi, virions; mg, midgut; Ub, ubiquitin. IP, immunoprecipitation; TCL, total cell lysate.

At 48 h post-infection (hpi), singly expressed P in Sf9 cells formed mobile punctate structures (Fig 4A). Immunoelectron microscopy showed that P was localized to granular inclusions in Sf9 cells expressing P (Fig 4B). Immunoelectron microscopy further showed that P and RdSina also localized to the viroplasm matrix in virus-infected leafhopper midgut epithelial cells (Fig 4C). Immunofluorescence microscopy showed that RdSina was recruited to the punctate structures of P in virus-infected leafhopper midgut epithelial cells (Fig 4D). Thus, RdSina acts as a RSMV restriction factor via interaction with viral viroplasm protein P.

We then investigated how RSMV-activated RdSina defended against viral propagation in *R. dorsalis*. The N-terminus of RdSina included a RING-Ubox domain (14–55 amino acids), thereby functioning as the E3 ligase (S6C, D Fig). In the presence of ubiquitin, E1, and E2, RdSina displayed a strong ligase activity as indicated by the generation of high molecular weight (MW) self-ubiquitinated protein bands (Fig 4E, 4F). In contrast, no signal was detected in the high MW ubiquitinated areas in the combinations lacking ubiquitin, E1, or E2, confirming that RdSina functions as an E3 ubiquitin ligase and can self-ubiquitinate (Fig 4E, 4F). To investigate whether RdSina can directly mediate P or NSs2 ubiquitination, *in-vitro* ubiquitination assays were performed using P or NSs2 as a substrate. Western blot assays showed that the polyubiquitinated P was generated in the presence of ubiquitin, E1, E2, and RdSina (Fig 4G, 4H), while NSs2 was not ubiquitinated in the presence of ubiquitin, E1, E2, and RdSina (S11A, B Fig). Thus, RdSina can ubiquitinate P but not NSs2 *in vitro*.

To investigate the *in-vivo* ubiquitination modification of P in Sf9 cells, P-His, RdSina-Strep and ubiquitin-Flag were co-expressed in Sf9 cells in the presence of MG132. Subsequently, the substrate protein P-His was immunoprecipitated with anti-His beads from the extracted cellular proteins. Following the immunoprecipitation of P-His, detection with a Flag antibody revealed that the presence of RdSina-Strep visibly increased the accumulation of ubiquitin chains in the immunoprecipitated P-His (Fig 4I), indicating that P is polyubiquitinated by RdSina in Sf9 cells.

Many ubiquitin chains with different biological functions have been reported, where 48th amino acid lysine (K48)-linked polyubiquitination leads to proteasome-mediated degradation of substrate proteins [33,34]. Immunoprecipitation assay also showed that overexpression of RdSina markedly increased the presence of K48-linked polyubiquitination bands on P (Fig 4I), suggesting the K48-linked polyubiquitin contributes to the degradation of P.

To investigate the ubiquitination modification of P in insect vectors, RdSina and ubiquitin were co-microinjected into RSMV-infected *R. dorsalis* following treatment with MG132. The substrate protein P was then immunoprecipitated with P antibody from the extracted total proteins. After immunoprecipitation of P, detection with ubiquitin antibody indicated that the presence of RdSina significantly increased the accumulation of ubiquitin chains in the immunoprecipitated P (Fig 4J), suggesting that P is also polyubiquitinated by RdSina in *R. dorsalis*.

### RdBV NSs2 antagonizes RdSina-mediated ubiquitinated degradation of RSMV P

**Fig 5:**
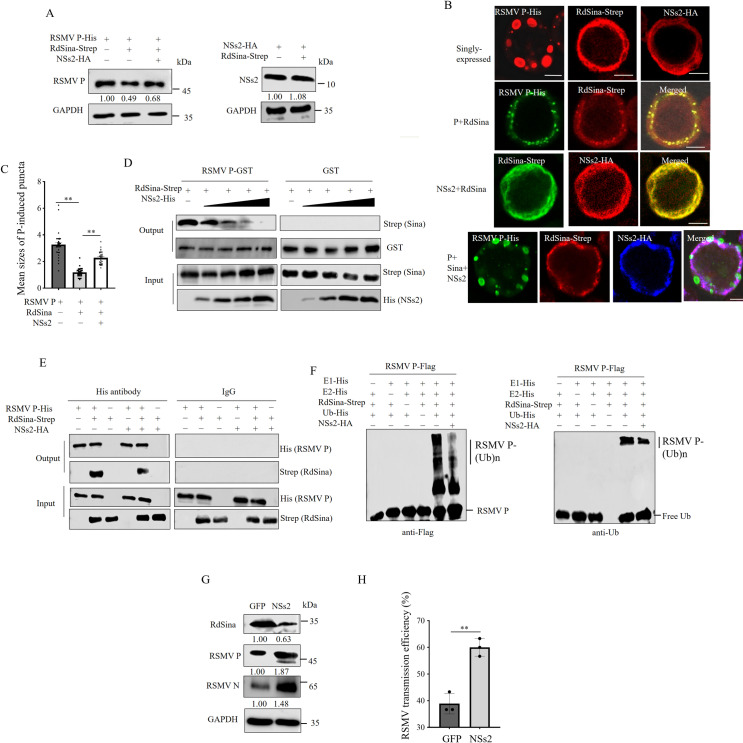
RdBV NSs2 competes with RSMV P in binding to RdSina and inhibits RdSina-mediated ubiquitination of RSMV P. (A) Western blot assay showing the accumulation levels of NSs2 or P in Sf9 cells singly expressing NSs2-HA or P-His, co-expressing RdSina-Strep with NSs2-HA or P-His, or triply expressing RdSina-Strep, P-His, and NSs2-HA. (B) Immunofluorescence assay showing the co-localization of RdSina, P, and NSs2 in Sf9 cells. Sf9 cells singly expressing RdSina-Strep, NSs2-HA, or P-His, co-expressing RdSina-Strep with NSs2-HA or P-His, or triply expressing RdSina-Strep, P-His, and NSs2-HA were immunolabeled with His-FITC (green), Strep-Alexa Fluor 647 (blue), or HA-rhodamine (red). Bars, 10 μm. (C) The mean sizes of 30 RSMV P-induced puncta in Sf9 cells were quantified using Image J software. The diameters of P-induced puncta are indicated as a dot plot, with the middle line representing the mean value. **, *p* < 0.01. (D) GST pull-down assay showing the competitive binding of RdSina by RdBV NSs2 or RSMV P using recombinant purified proteins. GST-P and Strep-RdSina were incubated with glutathione-Sepharose beads, then His-NSs2 was added to the beads. When the amounts of His-NSs2 were increased, the binding between GST-P and Strep-RdSina was decreased. (E) Competitive interactions for RdSina by RdBV NSs2 or RSMV P, as shown by Co-IP assay. Total proteins from Sf9 cells expressing RdSina-Strep and P-His were incubated with His or IgG antibody, then RdBV NSs2-HA was added to the mixture. When NSs2-HA was added, the binding between RdSina-Strep and P-His was decreased. (F) *In-vitro* ubiquitination assay showing that NSs2 inhibits RdSina-mediated ubiquitination of P. NSs2-HA or P-Flag and NSs2-HA together were used as substrates for the assay. Flag antibody was used to detect Flag-P, and ubiquitin (Ub) antibody was used to detect ubiquitination. (G) Western blot assay showing the effects of microinjection of RdBV NSs2 on the accumulation of RdSina and RSMV N and P in single RSMV-positive leafhoppers. (H) Efficiencies of RSMV transmission to rice seedlings by single RSMV-positive leafhoppers after the microinjection of GFP or NSs2, as calculated by the percentage of RT-PCR-positive plants out of the total number of tested plants. Data are presented as means (± SD) of three replicates, and each replicate contains 30 insects (two-tailed t test). *, *p* < 0.05. The proteins in A and G were detected by western blot assay using indicated antibodies. The relative intensities of the bands for these proteins are shown below using Image J. The data represent three biological replicates.

How RdBV suppressed the RdSina-mediated ubiquitinated degradation of P was also investigated. Western blot assay showed that RdSina expression led to the reduced accumulation of P in Sf9 cells co-expressing RdSina and P; however, the reduced accumulation level of P could be partially restored by the presence of NSs2 in Sf9 cells triply expressing NSs2, P and RdSina (Fig 5A). Immunofluorescence microscopy showed that the large punctate structures, formed by singly expressed P, were observed in Sf9 cells (Fig 5B). In contrast, singly expressed RdSina and NSs2 showed diffused distribution (Fig 5B). P and RdSina were co-localized in numerous small punctate structures in Sf9 cells co-expressing P and RdSina, whereas NSs2 and RdSina remained diffusely distributed in Sf9 cells co-expressing NSs2 and RdSina (Fig 5B). Furthermore, the sizes of the punctate structures of P in Sf9 cells triply expressing NSs2, P, and RdSina were observed to be larger than those in Sf9 cells co-expressing P and RdSina (Fig 5B, 5C). Significantly, the punctate structures of P in Sf9 cells triply expressing NSs2, P, and RdSina were observed to be dissociated from both NSs2 and RdSina (Fig 5B). The average sizes of the 30 punctate structures of P tested were approximately 3.27 μm when singly expressed, 1.18 μm when co-expressed with P and RdSina, and 2.28 μm when triply expressed with NSs2, P, and RdSina, as shown in Fig 5C. Thus, NSs2 can neutralize RdSina’s ability in mediating P degradation, ultimately promoting the formation of P-induced puncta.

Because both P and NSs2 can interact with RdSina, the competitive interaction between P-RdSina and NSs2-RdSina was investigated. GST Pull-down assay showed that the more NSs2 was present, the less RdSina binding to P occurred (Fig 5D). Co-IP assay showed that the presence of NSs2 levels led to the decreased RdSina levels binding to P in Sf9 cells (Fig 5E). *In-vitro* ubiquitination assay showed that NSs2 expression decreased RdSina-mediated polyubiquitination of P (Fig 5F). Microinjection of NSs2 into singly RSMV infected *R. dorsalis* decreased the accumulation level of RdSina but increased the accumulation level of P (Fig 5G), thereby promoting RSMV transmission efficiency (60%, average of 18/30, 19/30, and 17/30 plants), compared with 38.89% (average of 13/30, 11/30 and 11/30) after GFP injection (Fig 5H). Thus, NSs2 competes with P for binding to RdSina, and thus NSs2 potentially antagonizes RdSina-mediated ubiquitinated degradation of P, explaining why RdBV could enhance RSMV propagation and transmission in *R. dorsalis*.

### RdBV NSs2 enters the cellular nucleus to inhibit the transcription of RdSina

**Fig 6:**
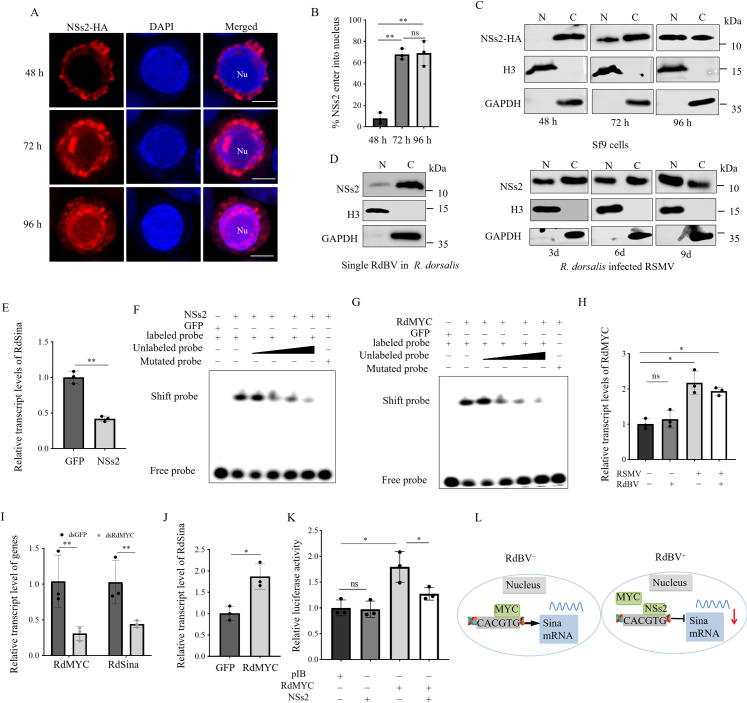
RdBV NSs2 suppresses the transcription of RdSina. (A) Immunofluorescence microscopy showing the distribution of NSs2 in Sf9 cells. Sf9 cells expressing NSs2-HA at different hpi were immunolabeled with HA-rhodamine (red) and DAPI (blue). Nu, nucleus. Bars, 10 μm. (B) The mean percentages of Sf9 cells with NSs2 in the nuclei. Data are presented as means (± SD) of three independent biological replicates and each replicate contains 30 cells (two-tailed t test). **, p < 0.01; ns, not significant. (C and D) Nuclear-cytoplasmic separation assay showing the nuclear (N) and cytoplasmic (C) distribution of RdBV NSs2 in Sf9 cells expressing NSs2-HA at different hpi (C), or in single RdBV-positive leafhoppers and RSMV-infected RdBV-positive leafhoppers at 3, 6, and 9 d (D). H3 and GAPDH antibodies reacted with the proteins of the nucleus and cytoplasm, respectively. (E) RT-qPCR assay showing the transcript levels of RdSina in RdBV^−^RSMV^−^ *R. dorsalis* individuals after microinjection of purified GFP or NSs2 proteins. (F and G) EMSA showing the binding ability of purified NSs2 (F) or RdMYC (G) to Cy5-labeled probes of RdSina promoter. Shift probes showing the binding of NSs2 or RdMYC to the Cy5-labeled probe of the RdSina promoter (CACGTG”-containing sequences). The unlabeled probe with the increased concentrations was added as a competitor. The mutated probe is a probe with “CACGTG”-containing sequences (CACGTG mutated to CAAAAA). (H) RT-qPCR assay showing the relative transcript and protein levels of RdMYC in RdBV^−^RSMV^−^, RdBV^+^RSMV^−^, RdBV^−^RSMV^+^ and RdBV^+^RSMV^+^ *R. dorsalis* individuals. (I) RT-qPCR assay showing the relative transcript levels of RdMYC and RdSina in dsGFP- or dsRdMYC-treated RSMV^−^RdBV^−^ *R. dorsalis* individuals. (J) RT-qPCR assay showing the transcript levels of RdSina in RdBV^−^RSMV^−^ *R. dorsalis* individuals after microinjection with purified GFP or RdMYC. (K) NSs2 inhibits the transcriptional activity of RdMYC. The pGL3-RdSina promoter was transfected with RdMYC alone, or NSs2 alone, or RdMYC together with NSs2 into Sf9 cells. The cells were harvested at 60 h after transfection, and the luciferase activities were measured by normalizing to the REN signals. (L) Proposed model showing that RdBV NSs2 inhibits the binding of RdMYC to the transcription factor-binding sites of the RdSina promoter. Data in E, H-K are presented as means (± SD) of three independent biological replicates and each replicate contains 30 insects (two-tailed t test). *, *p* < 0.05; **, *p* < 0.01; ns, not significant.

Previous studies reported that NSs proteins of bunyaviruses entered the nucleus and inhibited the transcription of some immune genes [35,36]. In Sf9 cells singly expressing NSs2, immunofluorescence microscopy showed that NSs2 initially distributed throughout the cytoplasm at 48 hpi and then entered the nucleus from 72 hpi (Fig 6A, 6B). The nuclear-cytoplasmic separation experiment confirmed the exclusive presence of RdBV NSs2 in the cytoplasm at 48 hpi, and in both the cytoplasm and nucleus at 72 hpi (Fig 6C). Similarly, the nuclear-cytoplasmic separation experiment showed the presence of NSs2 in the nucleus and cytoplasm of RdBV-positive *R. dorsalis* (Fig 6D). Western blot analysis using the nuclear and cytoplasmic fractions of RSMV-infected *R. dorsalis* further demonstrated the gradual accumulation of NSs2 in the nucleus as RSMV infection progressed (Fig 6D). Therefore, NSs2 seems to be translocated into the nucleus as RSMV infection continues, indicating the functional compartmentalization of NSs2 at different phases of RSMV infection (Fig 6D). RT-qPCR showed that RdSina was down-regulated in *R. dorsalis* after microinjection of NSs2 (Fig 6E), suggesting that NSs2 is involved in the negative regulation of RdSina expression.

Four possible transcription factor binding sites in the RdSina promoter regions were predicted using the BDGP (http://www.fruitfly.org) (S12A Fig). Electrophoretic mobility shift assay (EMSA) showed that RdBV NSs2 directly bound to the RdSina promoter sequences containing “CACGTG” (Fig 6F). Previous works have reported “CACGTG”-containing sequences to be bound by MYC oncogene transcription factor in mammalian [37]. The full-length ORF of *R. dorsalis* MYC (RdMYC) contained 1,512 nt which encoded 503 amino acid, and had a basic helix-loop-helix leucine-zipper (bHLHzip)_Myc motif (S12B Fig). EMSA assay confirmed that RdMYC also bound to the RdSina promoter sequences containing “CACGTG” (Fig 6G). However, NSs2 and RdMYC did not bind to the mutated sequences of “CACGTG” (CACGTG had been mutated to CAAAAA) (Fig 6F, 6G). The RdMYC transcript level was not changed significantly in single RdBV-positive individuals, but was significantly up-regulated in single RSMV-positive or RdBV and RSMV co-infected *R. dorsalis* (Fig 6H). Moreover, the knockdown of RdMYC expression by microinjection of dsRNAs targeting RdMYC (dsRdMYC) in RdBV-negative *R. dorsalis* effectively decreased the transcription of RdSina (Fig 6I). However, the transcription of RdSina was up-regulated in *R. dorsalis* after microinjection of RdMYC (Fig 6J), implying that RdMYC positively regulates RdSina expression.

Dual luciferase assay was performed to investigate whether the transcription factor RdMYC directly activated the transcription of RdSina. Sf9 cells were co-transfected with the RdMYC expression vector and the luciferase reporter construct that harbored the 2-kb promoter region of RdSina (RdSinapro-Luc). The activity of the RdSinapro-Luc reporter increased by 1.7-fold compared to cells transfected with empty expression vectors (Fig 6K). The activity of the RdSinapro-Luc reporter did not change by the expression of NSs2 alone, compared to cells transfected with empty expression vectors (Fig 6K). Interestingly, the reporter activities of RdSinapro-Luc were markedly inhibited by the co-expression of RdMYC and NSs2, compared with the single expression of RdMYC (Fig 6K). The competitive interaction assay showed that the more NSs2 was present, the less RdMYC binding to RdSina promoter occurred (S13 Fig). Thus, the binding of the RdSina promoter to NSs2 could suppress RdMYC-activated RdSina expression. Together, RSMV promotes the transcription of RdSina by activating the RdMYC, and NSs2 directly antagonizes the binding of RdMYC to the RdSina promoter to inhibit the transcription of RdSina.

## Discussion

**Fig 7:**
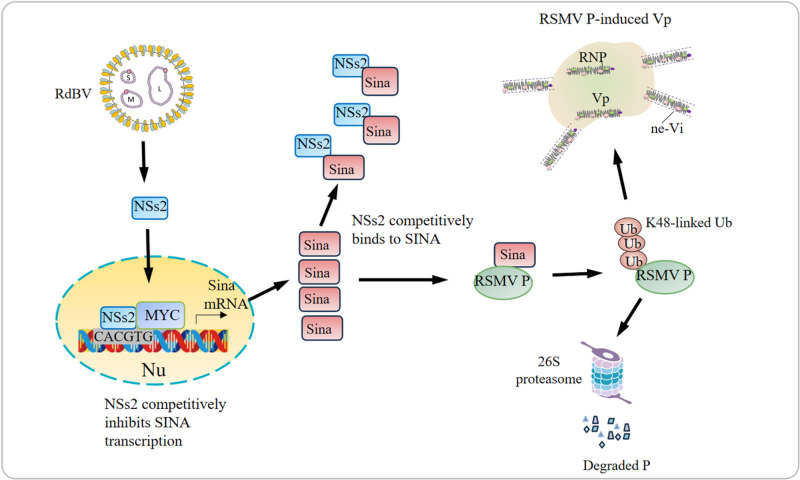
Proposed model showing that RdBV NSs2 antagonizes RdSina-mediated RSMV P degradation, thereby promoting RSMV propagation in co-infected insect vectors. RdSina attenuates the formation of RSMV P-induced viroplasm by promoting its degradation through the 26S proteasome. Also, RdBV NSs2 obstructs the function of RdSina by suppressing the transcription of RdSina and competing with RSMV P for binding to RdSina. Vp, viroplasm; RNP, ribonucleoprotein. ne-Vi: non-enveloped virions.

Multiple infections with insect symbiotic viruses and arboviruses are common in nature, and recent studies have shed light on the influence of insect symbiotic viruses on the transmission of arboviruses by insect vectors [1–3,38]. However, the molecular mechanisms underlying their direct synergistic interaction remain elusive. In current study, we find that an insect symbiotic bunyavirus (RdBV) enhances the transmission efficiency of RSMV by *R. dorsalis* in the field. We identify an E3 ubiquitin ligase Sina of *R. dorsalis*, RdSina, which directly interacts with and mediates the ubiquitin-dependent degradation of the RSMV phosphoprotein (P) through the 26S proteasome pathway. This process inhibits RSMV P-induced viroplasm formation, establishing RdSina as a viral restriction factor (Fig 7). Meanwhile, RdBV-encoded nonstructural protein NSs2 obstructs the function of RdSina by suppressing the transcription of RdSina and competing with RSMV P for binding to RdSina (Fig 7). This study provides the first report of Sina-mediated direct synergistic interaction between an insect symbiotic virus and a rice arbovirus, ultimately enhancing arbovirus transmission. Generally, Sina acts as an E3 ubiquitin ligase that targets specific proteins for degradation in diverse organs [39,40]. Our finding reveals a new regulatory mechanism of Sina expression by virus-encoded proteins and highlights a new function of Sina in mediating the degradation of viral replication proteins.

Rhabdoviruses comprise a large family whose collective host range includes vertebrates, invertebrates, and plants [27,41]. For instance, rabies virus causes lethal encephalitis, resulting in approximately 50,000 human deaths annually [37]. Plant rhabdoviruses, transmitted by insect vectors in a persistent-propagative manner, lead to significant agricultural losses [22,23,42]. The viroplasm serves as the site for viral replication and the assembly of progeny virions during rhabdovirus propagation, making viroplasm-induced viral proteins to act as the key targets for screening antiviral molecules. Rhabdovirus P is essential for viroplasm matrix formation, and recruits viral N and L to form the RNP cores within viroplasm matrix, thereby serving as the optimal target for antiviral defense [43,44]. RdSina contains a canonical C3-H-C4 type RING finger domain, which facilitates the transfer of ubiquitin chains onto targeted proteins [33]. In this study, we show that RdSina can self-ubiquitinate and mediate RSMV P polyubiquitination through the K48-linked ubiquitin chains, leading to P ubiquitinated degradation and inhibiting P-induced viroplasm formation. RSMV infection activates RdSina expression to control the excessive propagation of RSMV, thus facilitating viral persistent infection in insect vectors. Thus, RdSina acts as a restrictive factor for RSMV transmission by insect vectors. We deduce that Sina-mediated rhabdovirus P protein degradation is a potential conserved antiviral strategy exploited by various rhabdoviruses.

RdBV is a member of the genus *Cicadellivirus*, family *Phasmaviridae*, order *Bunyavirales*. Bunyaviruses in the *Phasmaviridae* family are arthropod-specific and are commonly found in various insects such as leafhoppers, mosquitoes, and psyllids [45–47]. The close phylogenetic relationship between insect-infecting phasmaviruses and their plant-infecting counterparts, such as tospoviruses and phenuiviruses, implies a potential common viral origin. However, bunyaviruses are non-replicative in plants, indicating the presence of host-specific barriers that restrict phasmaviruses to arthropods [48]. Although the vertical transmission rate of RdBV within *R. dorsalis* population is not 100%, rice plants might act as passive vectors for the horizontal transmission of RdBV, finally ensuring the effective spread of RdBV in nature. More importantly, RdBV promotes insect reproduction, thereby benefiting *R. dorsalis* population expansion, which explains why RdBV is an insect-specific symbiotic microorganism.

The genomes of phasmaviruses contain additional small ORFs besides the canonical ones currently known, and RdBV encodes small NSs1, NSs2, and NSs3 besides N. The small ORF-encoded proteins have been extensively reported to play important roles in the regulation of virus-host interactions [49–51]. In this study, we find that NSs2 has a dual function in decreasing the transcription level of RdSina and competitively inhibiting the ability of RdSina to bind to RSMV P, thus disrupting the RdSina-mediated degradation of RSMV P. A binding motif, RPVAxVxPxxR, for drosophila and mammalian Sina has been defined. The most conserved residues in this motif appear to be VxP domain, and indeed mutagenesis of both of these residues abrogates Sina binding [52]. Interestingly, P has VxP (242-VAP-244) motif while NSs2 not, which clarified why P is ubiquitinated while NSs2 not. Previous research has shown that NSs proteins of bunyaviruses can be found in both the nucleus and cytoplasm of infected cells, where they suppress host immune responses by inhibiting gene transcription [35,36]. For instance, the NSs protein of Rift Valley fever virus (a *phlebovirus* in the *Phenuiviridae* family within order *Bunyavirales*) suppresses cellular transcription by interacting directly with the transcription factor TFIIH [36]. Similarly, we find that RdBV NSs2 can enter the cellular nucleus and bind to RdSina promoter. The pairwise identity between NSs2 and NSs of tospoviruses ranged from approximately 20.45 to 21.74% (S1 Table), which suggest that Nss2 is genetically distinct. We further found that NSs2 has many Leucine-rich repeats such as LLLSH, LLSCLTH, RLWLQ, RLCCLLPPL. Previous studies reported that leucine-rich repeats may mediate protein dimerization, which is characteristic of many DNA-binding proteins [53]. Notably, we identify a new transcription factor MYC, which could bind to RdSina promoter, thus positively regulating RdSina transcription. Importantly, NSs2 effectively suppresses the transcriptional activity of MYC, potentially by competitively binding to the same sequences of the RdSina promoter. In a study of rice reovirus and its leafhopper vector, it was found that the insect ribosome-rescuer Pelo-Hbs1 complex, expressed on the sperm surface, mediates paternal transmission of arboviruses. The interaction between the RING domain of an E3 ubiquitin ligase and Pelo on sperm promotes the E3-mediated ubiquitination and degradation of Pelo via the 26S proteasome pathway. This degradation process also leads to the breakdown of its binding partner, Hbs1. Notably, Pns11 competes with Pelo for binding to the E3 ligase, thereby antagonizing the E3-mediated degradation of the Pelo-Hbs1 complex and facilitating paternal virus transmission [54]. We speculated that RdSina may not only target P but also act on sperm proteins to inhibit the vertical transmission of RdBV. In turn, RdBV appears to suppress RdSina to enhance its own vertical transmission. Thus, we report a previously undescribed function of a symbiotic virus in disturbing Sina-mediated ubiquitinated degradation of a viral replication protein.

Our results reveal that RSMV and RdBV have formed the cooperative interactions in insect vectors. However, the mechanism by which RSMV enhances RdBV propagation remains unknown. Previous study demonstrated that RSMV N serves as the effector to attenuate hemolymph melanization and facilitate viral persistent propagation in its insect vector [55]. Thus, RSMV can remodel antiviral response to benefit viral propagation, which may also explain why RdBV becomes highly efficient competence with RSMV and RdBV co-infected *R. dorsalis*.

For the first time, we reveal Sina-mediated direct synergistic interaction between an insect symbiotic bunyavirus and a rice rhabdovirus. Insect bunyaviruses in the *Phasmaviridae* family are prevalent in sap-sucking insects such as leafhoppers, psyllids and mosquitos [45–49]. Potentially, multiple infections of symbiotic bunyaviruses and arboviruses in insect vectors are common in nature. Moreover, the ubiquitination-proteasome system is a well-studied intracellular protein degradation pathway [31,32]. We thus anticipate that insect bunyaviruses-mediated the down-regulation of Sina expression is a conserved strategy used by more arboviruses to facilitate persistent transmission in nature.

## Materials and methods

### Identification, validation, and analyses of RdBV genome sequence

*R. dorsalis* populations used for this experiment were collected from rice fields in Guangdong Province, China, and were continuously reared on rice seedlings at 25 ± 3°C with a 16:8 h light/dark period and 40–60% relative humidity in the lab. High-throughput sequencing was performed for identification of insect-specific viruses from *R. dorsalis* population as described previously [1]. Thirty adults of *R. dorsalis* were collected and total RNAs were extracted using TRIzol Reagent according to the manufacturer’s instructions. RNA-seq library was constructed and sequenced using an Illumina NovaSeq 6000 platform in Novogene Co., Ltd, China. Low-quality reads and adapter sequences were removed from the raw reads to obtain clean reads. The clean reads were assembled and analyzed using BLASTx searches in the nonredundant protein database available in NCBI. BLAST results were then checked carefully to screen potential viral sequences.

A novel segmented single-stranded RNA virus was screened and then tentatively named RdBV. To confirm the existence of RdBV genome sequence discovered in the transcriptomes of *R. dorsalis*, ten specific primer pairs were designed using Primer Premier 5 (Premier, Canada) to amplify the overlapping fragments of about 1,000 nt throughout the contig sequence (S2 Table). The PCR products were cloned into the pTOPO vector (Genestar, China) for sequencing (Shanghai Sangon Biotech, China). The 5′ and 3′ RACE (rapid amplification of cDNA ends) was performed to verify the 5′ and 3′ terminal sequences using the SMART RACE cDNA amplification Kit (Clontech, California, USA). The primers used in RT-PCR and RACE-PCR were listed in S2 Table.

Full length of RdBV genome sequence was analyzed by NCBI ORF finder online (https://www.ncbi.nlm. nih.gov/orffinder). Secondary structure analysis was performed using RNAfold web server (http://rna.tbi.univie.ac.at//cgibin/RNAWebSuite/RNAfold.cg). To represent nucleotide bias at each position of the RNA sequences, we used the 20 nt from the 5′ and 3′ ends as input in WebLogo (v3.0). Phylogeny was analyzed based on comparison of RdRp amino acid sequences with members of family *Phasmaviridae* order Bunyavirales using MEGA 7 software. For phylogenetic tree reconstructions, maximum likelihood (ML) with P-distance was applied. The reliability of the ML trees was estimated by calculating bootstrap confidence limits based on 1,000 replicates.

### Insects, viruses, and antibodies

To establish the RdBV positive (RdBV^+^) or negative (RdBV^—^) *R. dorsalis* population, the pairs of one female and one male were put in glass tubes containing one rice seedling to produce offspring. Whether the parents were infected with RdBV or not was confirmed by RT-PCR assay. The offspring produced by RdBV-positive or negative parents were reared to establish RdBV-positive or negative population. The primers used were listed in S2 Table.

RSMV-infected rice plants were originally collected from Luoding city, Guangdong Province, China and maintained on rice plants via transmission by *R. dorsalis*. Rabbit polyclonal antibodies against RdBV N and NSs2, RdSina, and RSMV N and P were prepared by Genscript Biotech Corporation, Nanjing, China. Specific antibodies against RdBV N and NSs2, and RSMV N and P were conjugated to rhodamine or fluorescein isothiocyanate (FITC) to generate RdBV N-rhodamine, RdBV NSs2-rhodamine, RSMV N-FITC, and RSMV P-FITC, respectively. RdSina antibody was conjugated to Alexa Fluor 647 or rhodamine to generate RdSina-Alexa Fluor 647 or RdSina-rhodamine. Mouse or rabbit antibodies against GST or His, histone H3, HA, Flag, or Strep were purchased from Transgene Biotech (China). The actin dyes Alexa Fluor 647 phalloidin and the nuclear dye 4’,6-diamidino-2-phenylindole (DAPI) were purchased from Thermo Fisher Scientific.

### Acquisition and transmission rates of RSMV by *R. dorsalis*

To investigate how RdBV affected the acquisition and transmission of RSMV by *R. dorsalis*, 200 seconds-instar RdBV-positive or negative *R. dorsalis* individuals were allowed to feed on RSMV-infected rice plants for 2 days. Subsequently, insects were transferred to healthy rice plants. After 10 days, *R. dorsalis* individuals were placed in glass tubes that contained a single rice seedling and then were kept for 2 days. The insects were collected, and virus acquisition was assessed by RT-PCR assay. The plants inoculated with confirmed viruliferous insects were subjected to RT-PCR assay after 20 days. The transmission efficiency of RSMV by *R. dorsalis* was calculated.

### The prevalence of RdBV and RSMV in the field populations

To investigate the incidences of RdBV and RSMV in field populations, *R. dorsalis* samples were collected from Luoding field in 2021–2023. More than 100 individuals were tested for RdBV or RSMV from each field population via RT-PCR assay.

### Vertical transmission of RdBV

Four mating combinations of lab-reared *R. dorsalis* population were conducted as follows: (i) infected virgin female × infected male; (ii) infected virgin female × uninfected male; (iii) uninfected virgin female × infected male; and (iv) uninfected virgin female × uninfected male. In each combination, 50 newly emerged females and 50 newly emerged male adults were mated one to one in glass tubes containing one rice seedling for three days. The rice seedlings were replaced each day to avoid viral acquisition from rice plants. The females and males were then killed and tested by RT-PCR assay to confirm the uninfected or infected status. The eggs laid by these four combinations were harvested at seven days post oviposition by dissecting the rice seedlings and were individually placed on a piece of water-soaked filter paper in petri dishes at 25 ± 3 °C. After egg eclosion, offspring were individually fed on new rice seedlings, and the infection of RdBV was tested by RT-PCR assay. Each replicate contains 50 offspring from each mating combination and the primers used in RT-PCR assays were shown in S2 Table.

### Effect of RdBV infection on *R. dorsalis* fitness

Fifth-instar nymphs of *R. dorsalis* from RdBV-negative and positive colonies were individually reared with one healthy rice seedling in a glass tube. The growth of each leafhopper was monitored at 12-h intervals until the end of adult life to measure adult longevity. The longevity of 50 RdBV-negative and positive female and male adults was analyzed, and the experiment was replicated three times.

To examine the effect of RdBV infection on *R. dorsalis* offspring, two mating combinations from the lab-reared *R. dorsalis* colonies were conducted as follows: (i) infected virgin female ×infected male; and (ii) uninfected virgin female × uninfected male. In each combination, 10 newly emerged females and 10 newly emerged male adults mated one to one in glass tubes containing one rice seedling for 3 days. At seven days post oviposition, the eggs were collected, and the number of eggs were recorded. Then, 50 eggs were randomly collected from each combination and monitored at 12-h intervals until nymph emergence to determine the duration of egg development. Three independent biological replicates of each mating combination were conducted and analyzed.

### Immunofluorescence assay

To clarify the distribution of RdBV in different tissues of *R. dorsalis*, the intestine, salivary gland, and male or female reproductive organs were dissected from 30 RdBV-positive or negative *R. dorsalis* individuals. The samples were fixed in 4% (v/v) paraformaldehyde in PBS for 2 h, and then permeabilized in 2% (v/v) Triton-X (Sigma-Aldrich, T8787) for 1 h. The samples were then incubated with RdBV N-rhodamine (0.5 μg/μL) and the actin dye Alexa Fluor 647 phalloidin (0.1 μg/μL). Immunostained tissues were visualized using a laser scanning confocal microscope (LSCM, Leica TCS SPE).

To confirm the biological differences of RSMV infection between RdBV-positive and negative populations, second-instar nymphs of RdBV-positive and negative *R. dorsalis* individuals were allowed to feed on RSMV-infected rice plants for 2 days and then transferred to healthy rice seedlings. The intestines were fixed, permeabilized, immunolabeled with RdBV N-rhodamine or RSMV N-FITC, and then examined by confocal microscopy.

For visualizing the association of viruses and RdSina, the intestines were excised from 30 RSMV-negative and positive *R. dorsalis* individuals. The samples were successively fixed, permeabilized, immunolabeled with RdBV N-rhodamine, RSMV P-FITC, or RdSina-Alexa Fluor 647, and then examined by confocal microscopy.

### Immunoelectron microscopy

For immunoelectron microscopy, the excised intestines were fixed in 4% (v/v) paraformaldehyde in PBS for 3 h at 4ºC, sequentially dehydrated in 30%, 50%, 70%, 90% and 100% alcohol, and embedded in LR Gold Resin (Bioscience). The specimen was sectioned on an ultramicrotome (Leica), then incubated in blocking buffer (goat serum, 1:100) for 30 min. The sections were incubated with antibodies against RdSina, RdBV N, and RSMV P, and then incubated with 15-nm gold-conjugated goat-anti-rabbit IgG for 2 h. To observe the structure of RSMV P in Sf9 cells, the sections of Sf9 cells expressing GFP or RSMV P were incubated with RSMV P antibody, and then incubated with 15-nm gold-conjugated goat-anti-rabbit IgG for 2 h. The samples were then immunolabelled and observed with a transmission electron microscope (H-7650; Hitachi).

### RNA-seq analysis

To examine the expression profiles of *R. dorsalis* in response to RdBV infection, total RNAs extracted from RdBV-positive or negative *R. dorsalis* population were used for RNA-seq analysis. Differentially expressed genes were identified according to the transcripts per million reads (TPM) (log2[fold change] ≥ 1 or ≤ −1 and P ≤ 0.05). Furthermore, Kyoto Encyclopedia of Genes and Genomes (KEGG, http://www.genome.jp/kegg/) analyses were performed to identify differentially expressed genes that were significantly enriched in KEGG pathways.

### Y2H assay

The full-length ORFs of RdBV NSs2, NSs1, NSs3, N, G, and NSm, and RSMV P, G, P3, and P6 were individually cloned into the vector pGBKT7. The full-length ORFs of RdSina, and RSMV N and M were individually cloned into the vector pGADT7. Yeast strain AH109 was transformed with bait and prey plasmids pairings using the lithium acetate method with single‐stranded carrier DNA according to the manufacturer’s protocol (Dualsystems Biotech, Switzerland). Transformants were subsequently dropped on the DDO (SD/-Trp-Leu) and QDO (SD/-Trp-Leu-His-Ade/X-Gal) culture medium and incubated at 30°C for 3–5 days. The positive control pGBKT7–53/pGADT7-T and negative control pGBKT7-Lam/pGADT7-T were transformed in the same way. The primers used in Y2H were shown in S2 Table.

### GST pull-down assay

The ORFs of RdBV NSs2 and RSMV P were inserted into the vector pGEX-4T-3 to express RdBV NSs2-GST and RSMV P-GST, respectively. The ORF of RdSina was cloned into the vector pET28a (His tag) for expressing RdSina-His. Fusion proteins were expressed in the *Escherichia coli* strain BL21 by induction of 0.1 mM Isopropyl-D1-Thiogalactopyranoside at 16°C for 10 h. For GST pull-down assays, GST, GST-P, or GST-NSs2 was incubated with 50 µL glutathione Sepharose4B beads (GE Healthcare, USA) at 4°C for 2 h. The bead bound proteins were washed five times and then incubated with His-RdSina for 4 h at 4°C. After being centrifuged and washed five times, the mixed proteins were separated by SDS-PAGE gel electrophoresis and detected by western blot assay with His-tag and GST-tag antibodies.

Furthermore, competitive binding assay was performed to evaluate the binding affinities difference between P-RdSina and NSs2-RdSina. Briefly, GST-P and Strep-RdSina were incubated with GlutathioneSepharose4B beads for 4 h at 4°C, and the HA-NSs2 was subsequently added at different amounts to the beads and incubated for 2 h at 4°C. The bead bound proteins were washed with PBS, then separated and detected as described above.

### Co-IP assay

For the Co-IP assay, recombinant bacmids expressing His-P were co-transfected with recombinant bacmids expressing Strep-RdSina into Sf9 cells. After two days, the Sf9 cells were harvested for extraction of total proteins. His antibody was incubated with protein samples of the Sf9 cells expressing RSMV P and RdSina for 4 h at 4°C, and then RdBV NSs2 was added to the mixture. Then, protein A/G-agarose beads (Beyotime, Jiangsu, China) were added and incubated for 2 h at 4°C. After washing five times with lysis buffer, immunoprecipitated proteins were detected by western blot with Strep and His antibodies.

### Expression analysis of RdSina and RdMYC in *R. dorsalis* during RdBV or RSMV infection

To analyze the effects of RdBV or RSMV infection on the expression levels of RdSina in *R. dorsalis*, insect total RNAs and proteins were extracted from 30 RdBV and RSMV co-negative, RdBV-positive and RSMV-negative, RdBV-negative and RSMV-positive, or RSMV and RdBV co-positive *R. dorsalis* individuals. The relative transcript and protein expression levels of RdSina in different combination insect populations were detected by RT-qPCR and western blot assays. In the corresponding western blot assay, antibodies against RdSina, RdBV N, RSMV N, and GAPDH (0.5 μg/μL) served as the primary antibodies, and goat anti rabbit IgG-peroxidase (0.5 μg/μL) served as the secondary antibody. The relative transcript levels of RdMYC in different combination insect populations were also detected by RT-qPCR assays as above.

### Effect of *in-vitro* synthesized dsRNAs on viral infection and transmission rates in *R. dorsalis*

RNA inference was carried out to suppress the expression of related genes and confirm their functions on viral propagation and transmission by insect vectors. The T7 promoter with the sequence 5′-ATTCTCTAGAAGCTTAATACGACTCACTATAGGG-3′ was added to the forward and reverse primers to amplify a partial region of ~500–800 bp of RdSina, RdMYC, or GFP (S2 Table). The dsRNAs targeting RdSina (dsRdSina), RdMYC (dsRdMYC), or GFP (dsGFP) were synthesized according to the protocol for the T7 RiboMAX Express RNAi System kit (Promega, P1700).

To test the suppression of RdSina expression on RdBV or RSMV infection in *R. dorsalis*, RSMV and RdBV co-negative or RdBV-positive and RSMV-negative second-instar *R. dorsalis* individuals were fed on RSMV-infected rice plants for two days and then were microinjected with 23 nl dsRdSina (3 μg/μL) or with dsGFP (3 μg/μL) using an Auto-Nanoliter Injector (Drummond). The dsRNAs-treated leafhoppers were transferred to healthy rice seedlings and approximately 50 treated leafhoppers were collected at 4-day post-first access to diseased plants (padp). The total RNAs were extracted from each of leafhoppers, and RT-PCR assay was performed to confirm that they were viruliferous. Furthermore, total RNAs from 30 leafhoppers were extracted to estimate RdSina transcript levels and RdBV or RSMV titers by RT-qPCR assays. Total proteins were extracted from 50 leafhoppers to quantify the accumulation levels of RdSina, RdBV N, RSMV N, or P using western blot assays. To investigate the impact of RdSina on the transmission of RSMV by *R. dorsalis*, 200 dsRNAs-treated leafhopper individuals were placed in glass tubes that contained a single rice seedling for a 48‐h inoculation access period. The seedlings were then grown in a greenhouse. At 15 days post‐inoculation, the rice plants were surveyed for disease symptoms and subjected to RT-PCR testing for RSMV.

### Effects of microinjection of NSs2 on accumulation of RdBV and RSMV

To test the microinjection of NSs2 on accumulation of RSMV, second-instar *R. dorsalis* individuals were fed on RSMV-infected rice plants for two days and then were microinjected with 23 nl NSs2 or GFP (1 mg/ml) using an Auto-Nanoliter Injector. The protein-treated leafhoppers were transferred to healthy rice seedlings and approximately 50 treated leafhoppers were harvested at 4-day padp. Furthermore, total proteins from 30 leafhoppers were extracted to quantify the accumulation levels of RdSina, RSMV N, or P using western blot assays.

To test the microinjection of RdMYC or NSs2 on the transcript levels of RdSina, RSMV and RdBV co-negative second-instar *R. dorsalis* individuals were microinjected with 23 nL RdMYC, NSs2, or GFP (1 mg/ml) using an Auto-Nanoliter Injector. The protein-treated leafhoppers were transferred to healthy rice seedlings and approximately 50 treated leafhoppers were harvested at 4-day padp. Furthermore, total RNAs from 30 leafhoppers were extracted to estimate RdSina transcript levels by RT-qPCR assays. To investigate the impact of NSs2 on the transmission of RSMV by *R. dorsalis*, 200 microinjected leafhopper individuals were placed in glass tubes with a single rice seedling for a 48‐h inoculation access period. Subsequently, the seedlings were grown in a greenhouse. At 15 days post‐inoculation, the rice plants were surveyed for disease symptoms and subjected to RT-PCR testing for RSMV.

### Effects of proteasome inhibitor on the accumulation of RdBV and RSMV

RSMV and RdBV co-negative second-instar *R. dorsalis* individuals were allowed to feed on RSMV-infected rice plants for 2 days and then transferred to healthy rice seedlings for 1 day. Further, the leafhoppers were microinjected with MG132 (10 μm) or DMSO buffer. The protein accumulation levels of GADPH, and RSMV N and P in leafhoppers were determined at 6-day padp by western blot assays.

In addition, RdBV-positive and RSMV-negative second-instar *R. dorsalis* individuals were microinjected with MG132 (10 μm) or DMSO buffer, and then transferred to healthy rice seedlings for 6 days. The protein accumulation levels of GADPH, and RdBV N and NSs2 were tested by western blot assays.

Sf9 cells expressing RdBV NSs2 or RSMV P were treated by MG132 (10 μm) or DMSO buffer for 6 h and the protein accumulation levels of GADPH, and RdBV NSs2 or RSMV P were tested by western blot assays.

### Baculovirus expression assay

To confirm the relationship among RdSina, RdBV NSs2, and RSMV P in Sf9 cells, the baculovirus system was used to express RdSina, NSs2, and P. The ORFs of RdSina, NSs2, and P were inserted into vector pFAST‐T1 with Strep, HA, and His tag respectively using the designed primers in S2 Table. The plasmids were transformed into *E. coli* DH10Bac cells for transposition into the bacmids. Recombinant bacmids containing RdSina, NSs2, or P were transfected into Sf9 cells with Cellfectin II Reagent (Thermo Fisher Scientific, 10362100). After incubation for 48 h, the Sf9 cells grown on the coverslips were fixed, permeabilized, immunolabelled with His-FITC, HA-rhodamine, or Strep-Alexa Fluor 647, then processed for immunofluorescence microscopy.

### *In-vitro* ubiquitination assays

The ORFs of RdE1, RdE2, Rdubiquitin were cloned into the vector pET28a (His tag) for expressing RdE1-His, RdE2-His, RdUb-His. Recombinant proteins with His tag were expression in *E. coli* strain BL21 (DE3) and purified using Ni Sepharose (Genscript). For the E3 Ub ligase activity assay of the fusion proteins, purified Strep-RdSina was incubated with E1, E2, ubiquitin and 1.5 mL of 20 × reaction buffer (1 M Tris HCl, pH 7.5, 40 mM ATP, 100 mM MgCl2, 40 mM DTT, 600 mM creatine phosphate, and 1 mg/mL creatine phosphokinase). The reactions were incubated at 30°C for 1.5 h in a 30 μL reaction volume. After the reactions, proteins were separated by SDS-PAGE, followed by immunoblot analyses using antibodies against RdSina and ubiquitin for detection of Strep-RdSina and ubiquitin, respectively.

The ORFs of HA-NSs2 or Flag-P were cloned into the vector pET28a (His tag) for expressing HA-NSs2, Flag-P. HA-NSs2 or Flag-P were expression in *E. coli* strain BL21 (DE3) and purified using Ni Sepharose. To determine whether RdBV NSs2 and RSMV P could be ubiquitinated by RdSina, purified HA-NSs2 or Flag-P was incubated with the ubiquitination mixture. Mixtures were then subjected to SDS-PAGE and immunoblotting assay with anti-Flag, anti-HA, or anti-ubiquitin antibody for detection of Flag-P or HA-NSs2 substrate proteins.

### Immunoprecipitation assay for detecting RSMV P ubiquitination in Sf9 cells

Ubiquitination is a mode of protein regulation, and MG132, as a proteasome inhibitor, cannot inhibit the ubiquitination process itself, but it can inhibit the proteasome degradation process after ubiquitination, thereby indirectly promoting the accumulation of ubiquitination. In order to detect the ubiquitination of RSMV P in Sf9 cells, Flag-tagged ubiquitin (ubiquitin-Flag) and a mutant retaining only the 48th lysine site (ubiquitin-K48-Flag) were inserted into Fast-bac1 vector and then introduced into *E. coli* DH10Bac to construct the recombinant bacmids. Subsequently, Sf9 cells were co-expressed with P-His with ubiquitin-Flag/ubiquitin-K48-Flag, or triply expressed with P-His, RdSina-Strep, and ubiquitin-Flag/ubiquitin-K48-Flag. Subsequently, the cells were treated by MG132 (10 μm) for 6 h. Total proteins were extracted and P-His was immunoprecipitated using His antibody for 4 h at 4°C Protein A/G-agarose beads were added and incubated for 2 h at 4°C. After washing five times with lysis buffer, the polyubiquitination of P was detected with Flag antibody and the total cell lysate (TCL) was detected by GAPDH or His antibody through western blot assays.

### Immunoprecipitation assay for detecting RSMV P ubiquitination in *R. dorsalis*

To detect ubiquitination of RSMV P in *R. dorsalis*, RSMV and RdBV co-negative second-instar *R. dorsalis* individuals were allowed to feed on RSMV-infected rice plants for 2 days and then transferred to healthy rice seedlings for 1 day. The insect individuals were co-microinjected with ubiquitin and MG132 (10 μm), or with RdSina, ubiquitin, and MG132 (10 μm). The total insect proteins were extracted at 6-day padp and immunoprecipitated by P antibody for 4 h at 4°C. Protein A/G-agarose beads were added and incubated for 2 h at 4°C. After washing five times with lysis buffer, immunoprecipitated proteins were detected by western blot assays with ubiquitin antibody and the TCL was detected by GAPDH or P antibody.

### Nuclear-cytoplasmic separation assay

Nuclear and cytoplasmic proteins were extracted from 30 RdBV-positive or negative *R. dorsalis* individuals or Sf9 cells expressing NSs2 for 2–4 days using a Nuclear and Cytoplasmic Extraction kit (Beyotime). The samples were grinded and incubated with cytoplasmic extraction A and B reagents (volume ratio of 20:1) supplemented with a protease inhibitor cocktail (Thermo Fisher Scientific, Waltham, MA, USA) for 30 min. The homogenate was then centrifuged and the supernatant was retained as the first cytoplasmic protein fraction. Subsequently, the precipitate was resuspended by another 200 μL of cytoplasmic extraction reagent A. After incubating for 15 min, the homogenate was added with 10 μL of cytoplasmic extraction reagent B and centrifuged. The resulting supernatant was retained as the second cytoplasmic protein fraction. Both extracts were then mixed and used as cytoplasmic proteins. Subsequently, the precipitate was added with 50 μl of nuclear extraction reagent and vortexed for 30 min. At last, the supernatant was retained as the nuclear protein extract fraction after centrifugation. The cytoplasmic and nuclear proteins were detected by western blot assays. The H3 and GAPDH were used as reference proteins for nuclear and cytoplasmic proteins, respectively.

### Target prediction in the RdSina promoter regions by transcription factor

RdSina promoter sequence was regarded as the 2,000-bp sequence upstream of the transcription start site of each gene. We predicted the potential targets in the RdSina promoter regions by transcription factor using the BDGP (http://www.fruitfly.org). Four candidate targets were verified via EMSA.

### EMSA

Cy5-labelled RdSina promoter probe, or competitor probe and mutated probe (CACGTG mutated to CAAAAA) were synthesised by SYNA (China, Fuzhou). NSs2 or RdMYC were purified and incubated with RdSina promoter probe or competitor in 5 × binding buffer (1 M Tris HCl pH 7.5, 5 M NaCl, 1 M potassium chloride, 1 M magnesium chloride, 0.5 M EDTA pH 8.0, 10 mg/ml BSA). The reaction mixture was separated by PAGE and then scanned by Odyssey CLx (Li-Cor, Lincoln, NE, USA). The detailed probe information of the RdSina promoter was shown in S2 Table.

### Dual-luciferase reporter assay

The transcriptional activity of RdMYC or NSs2 was detected with luciferase reporter assay in transiently transfected Sf9 cells. About 2, 000-bp RdSina promoter sequences were amplified and then ligated into pGL3 vector (Promega, Madison, WI, USA). RdMYC or NSs2 was cloned and inserted into the expression vector pIBV5-His (Invitrogen, San Diego, CA, USA). The primers used were shown in S2 Table.

For the luciferase assay, Sf9 cells were seeded in a 12-well plate and co-transfected in the transfection mixture containing 1 μg of the RdSina promoter vector, 1 μg expression plasmid of RdMYC, and 0.02 μg of the internal Renilla luciferase control reporter plasmid pRL-CMV (Promega) per well using 4 μl of Cellfectin II Reagent (Promega) to identify the activity of RdMYC. The mixture containing RdSina promoter vector, pIBV5, and pRL-CMV were as control, and the mixture containing RdSina promoter vector, RdMYC-pIBV5, NSs2-pIBV5, and pRL-CMV were transfected to identify the function of NSs2 on RdMYC activity. At 48 h posttransfection, the cells were collected and processed with the dual-luciferase reporter assay kit (Vazyme, China) according to the manufacturer’s protocol. The Firefly and Renilla luciferase activities were measured by the FlexStation-3 microplate reader (Molecular Devices). The relative firefly luciferase activity normalized against the Renilla luciferase activity was shown as the means (± SD) of three independent transfections.

### Competitive binding assay between RdMYC-RdSina promoter and NSs2-RdSina promoter

Competitive binding assay was performed to evaluate the binding affinities difference between RdMYC-RdSina promoter and NSs2-RdSina promoter. Briefly, RdSina promoter-biotin and RdMYC were incubated with streptavidin beads (Beyotime) for 4 h at 4°C, and the NSs2 was subsequently added at different amounts to the beads and incubated for 2 h at 4°C. The bead bound proteins were washed with PBS and detected by western blot.

## Supporting information

S1 Fig Bioinformatics analysis of RdBV. (A) Conserved terminal sequences and segment-specific inverted repeats in the terminal regions of positive-sense strands of three genome segments of RdBV. Sequences adjacent to the conserved termini are inverted repeats and predicted to form secondary structures. (B) Terminal nucleotide sequences of RdBV three genome segments. Note the conservation of the 5′-TAGCAGCACGCTG and 3′-TCGTCGTACGAC terminal sequences among the genome segments. (C) Phylogenetic relationships of RdBV with members of the family *Phasmaviridae*, order Bunyavirales. The available amino acid sequences of RdRp amino acid sequences from RdBV and the counterparts were analyzed for construction of phylogenetic trees. The position of RdBV is indicated by a red star. Bootstrap values generated from 1,000 replicates are shown above each node. The scale bars indicate the evolutionary distance expressed as amino acid substitutions per site.
(TIF)

S2 Fig Immunofluorescence microscopy showing RdBV infection in different organs of *R. dorsalis.* Salivary glands, ovaries, or testes of RdBV-negative and positive *R. dorsalis* were dissected, immunostained with RdBV N-FITC (green) and actin dye phalloidin-rhodamine (red), and then observed by immunofluorescence microscopy. Panels ii, iv and vi are the enlarged images of the boxed areas in panels i, iii and v, respectively. Bars, 100 μm. Od, oviduct; Oo, oocyte; Pd, pedicel; Sp, sperm.
(TIF)

S3 Fig Effects of RdBV infection on the fitness of *R. dorsalis* adults and offspring. Mating combinations were established as follows: infected virgin female × infected male, and uninfected virgin female × uninfected male. (A) Effects of RdBV infection on the longevity of adults. Longevity of 50 RdBV-negative and positive female and male adults. Means (± SD) are shown from 50 insects, and represent three biological replicates (two-tailed t test). (B and C) Progeny egg number (B) and developmental duration (C) of different mating combinations. Data are presented as means (± SD) of three independent experiments of two mating combinations (two-tailed t test). *, *p* < 0.05; **, *p* < 0.01; ns, not significant.
(TIF)

S4 Fig RSMV accumulation level (A) and symptom (B) in rice plants infected by RdBV-negative and positive *R. dorsalis.* (TIF)

S5 Fig (A and B) RT-qPCR (A) and western blot (B) assays showing the transcript and protein levels of RdBV N in RSMV-positive and negative *R. dorsalis* population. (TIF)

S6 Fig Analysis of differentially expressed genes in RdBV-positive and negative *R. dorsalis.* (A and B) KEGG analysis of up-regulated (A) and down-regulated genes (B) in RdBV-positive *R. dorsalis* compared with RdBV-negative *R. dorsalis*. (C) Characterization of RdSina containing a RING-Ubox domain. (D) Illustration of the RdSina C3-H-C4 RING finger composition.
(TIF)

S7 Fig Y2H assays for testing the interactions among RdSina, RSMV-encoded proteins, and RdBV-encoded proteins. (A) Y2H assays for testing the interactions between RdSina with RdBV-encoded proteins. (B) Y2H assays for testing the interactions between RdSina with RSMV-encoded proteins. (C) Y2H assays for testing the interactions between RSMV-encoded proteins and RdBV-encoded proteins.
(TIF)

S8 Fig Y2H assays identifying the regions of RdSina required for interaction with RdBV NSs2 or RSMV P. (A) Schematic representation of the truncated mutants of RdSina. (B) Y2H assay for testing the interactions between RdSinaC (65–268 aa) and RdBV NSs2 or RSMV P.
(TIF)

S9 Fig Immunoelectron microscopy showing the viroplasm in the midgut epithelial cells of MG132 or DMSO-treated RSMV infected *R. dorsalis.* The mean number of viroplasms per midgut epithelial cell of MG132 or DMSO-treated RSMV infected *R. dorsalis* is shown in S9A (n = 20). Vi, virions. Vp, viroplasm. Bars: 200 nm.
(TIF)

S10 Fig RSMV accumulation level (A) and symptom (B) in rice plants infected by dsGFP or dsRdSina-treated *R. dorsalis.* (TIF)

S11 Fig RdBV NSs2 was not ubiquitinated by RdSina. HA-NSs2 was used as a substrate for the assay. HA antibody was used in the immunoblotting assay to detect HA-NSs2 (A), and ubiquitin antibody was used to detect His-ubiquitin (B).
(TIF)

S12 Fig Characterization of RdMYC and RdSina promoter sequences. (A) Transcription factor-binding sites prediction in RdSina promoter regions. Four probe sequences (P1, P2, P3, P4) in the RdSina promoter regions were predicted to contain the transcription factor binding motif. (B) Characterization of RdMYC containing the bHLHzip_Myc domain.
(TIF)

S13 Fig Competitive binding assay showing the binding affinities difference between RdMYC-RdSina promoter and NSs2-RdSina promoter. RdMYC and RdSina promoter were incubated with glutathione-Sepharose beads, then His-NSs2 was added to the beads. When the amounts of NSs2 were increased, the binding between RdMYC and RdSina promoter was decreased.
(TIF)

S1 Table Percentage identity of NSs2 in comparison with other proteins. (DOCX)

S2 Table List of oligonucleotide primers used in this study. (DOCX)

S1 Data The raw data used in the figures. (XLSX)
