# Supplementary figures and images for: An insect symbiotic virus promotes the transmission of a phytoarbovirus via inhibiting E3 ubiquitin ligase Sina
Source: PLoS Pathog. 2025 May 29;21(5):e1013178. doi: 10.1371/journal.ppat.1013178 (PMC12121772; doi:10.1371/journal.ppat.1013178)

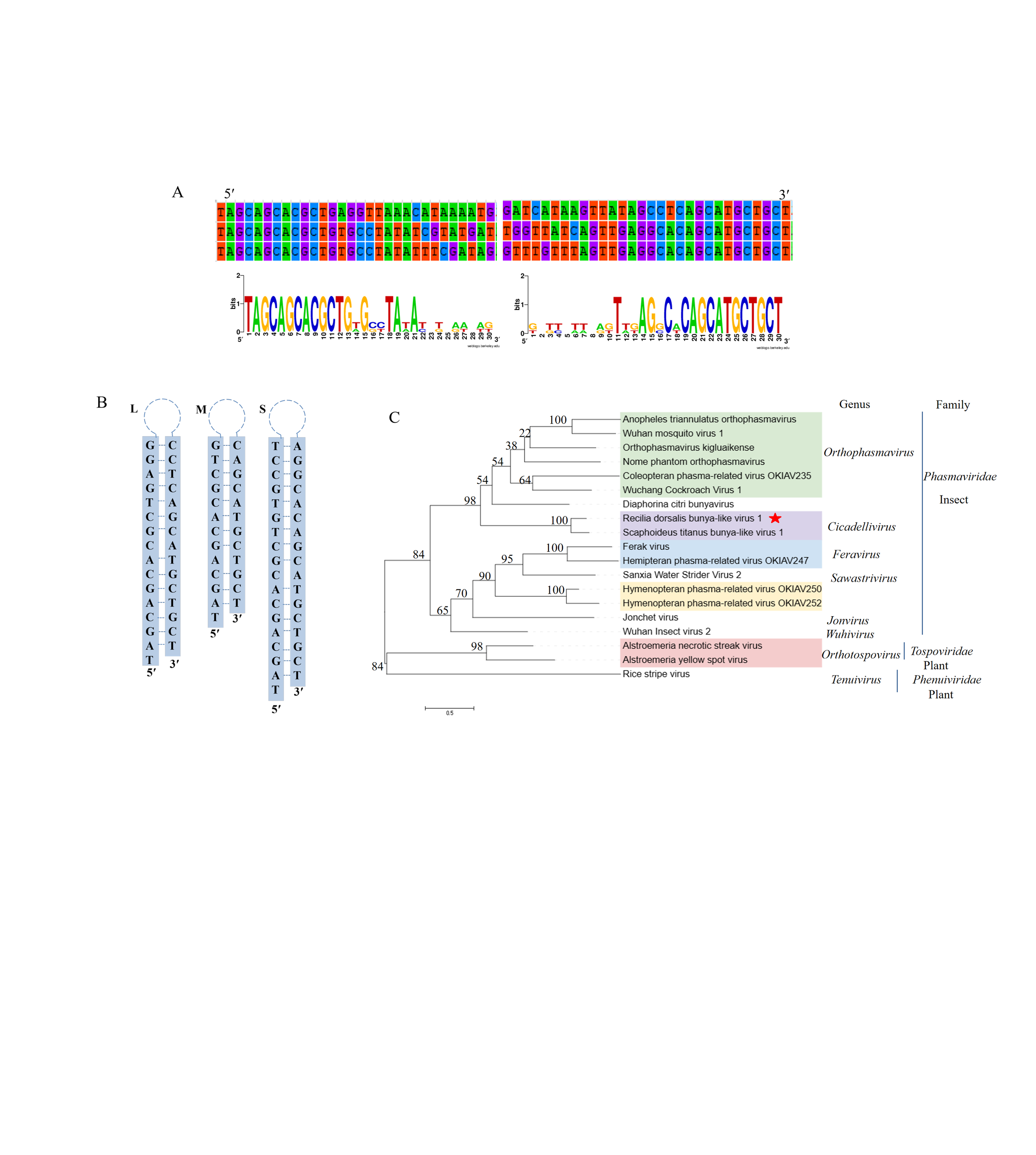

Supplement: S1 Fig — (A) Conserved terminal sequences and segment-specific inverted repeats in the terminal regions of positive-sense strands of three genome segments of RdBV. Sequences adjacent to the conserved termini are inverted repeats and predicted to form secondary structures. (B) Terminal nucleotide sequences of RdBV three genome segments. Note the conservation of the 5′-TAGCAGCACGCTG and 3′-TCGTCGTACGAC terminal sequences among the genome segments. (C) Phylogenetic relationships of RdBV with members of the family Phasmaviridae, order Bunyavirales. The available amino acid sequences of RdRp amino acid sequences from RdBV and the counterparts were analyzed for construction of phylogenetic trees. The position of RdBV is indicated by a red star. Bootstrap values generated from 1,000 replicates are shown above each node. The scale bars indicate the evolutionary distance expressed as amino acid substitutions per site. (TIF) [file ppat.1013178.s001.tif]

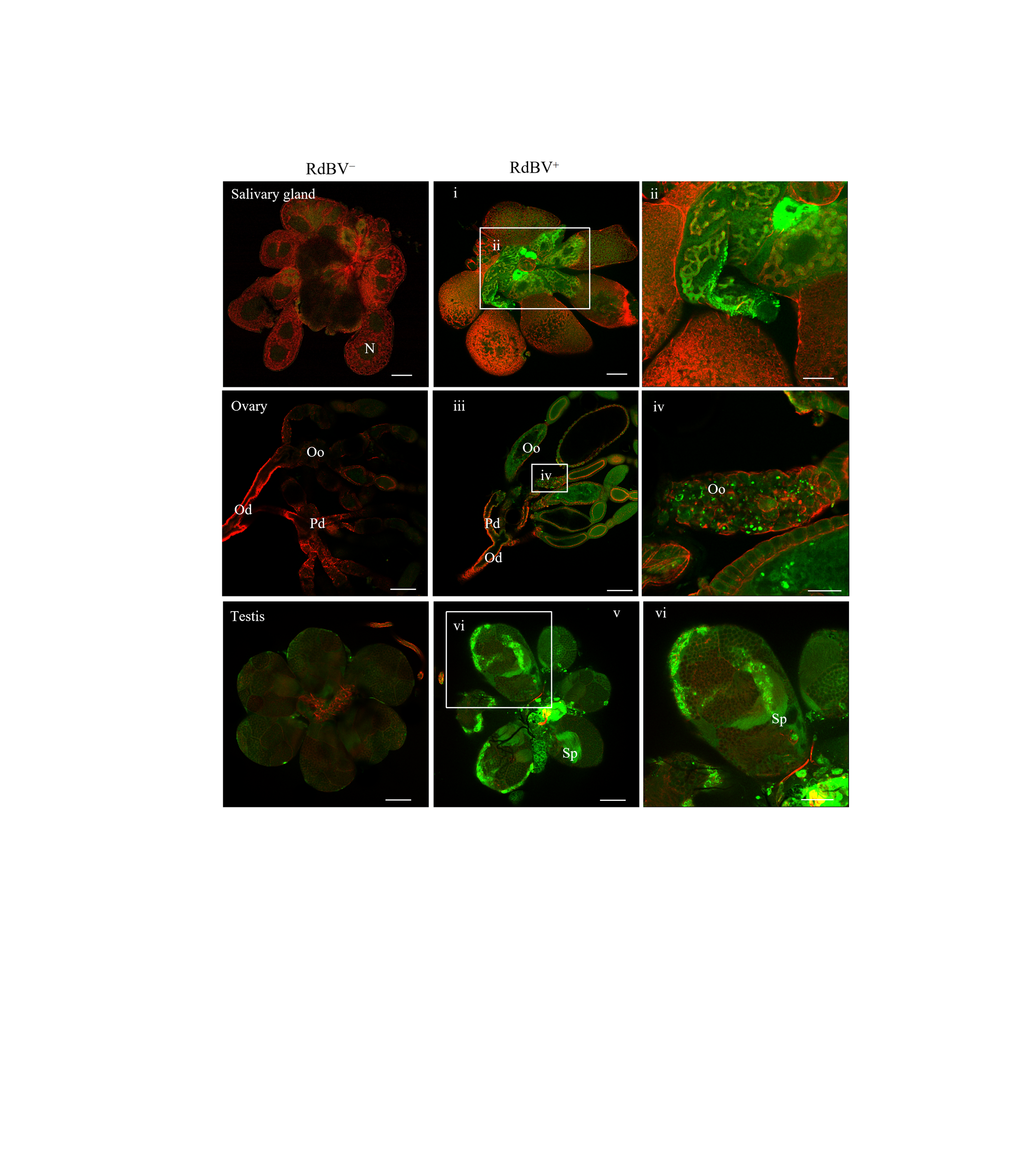

Supplement: S2 Fig — Salivary glands, ovaries, or testes of RdBV-negative and positive R. dorsalis were dissected, immunostained with RdBV N-FITC (green) and actin dye phalloidin-rhodamine (red), and then observed by immunofluorescence microscopy. Panels ii, iv and vi are the enlarged images of the boxed areas in panels i, iii and v, respectively. Bars, 100 μm. Od, oviduct; Oo, oocyte; Pd, pedicel; Sp, sperm. (TIF) [file ppat.1013178.s002.tif]

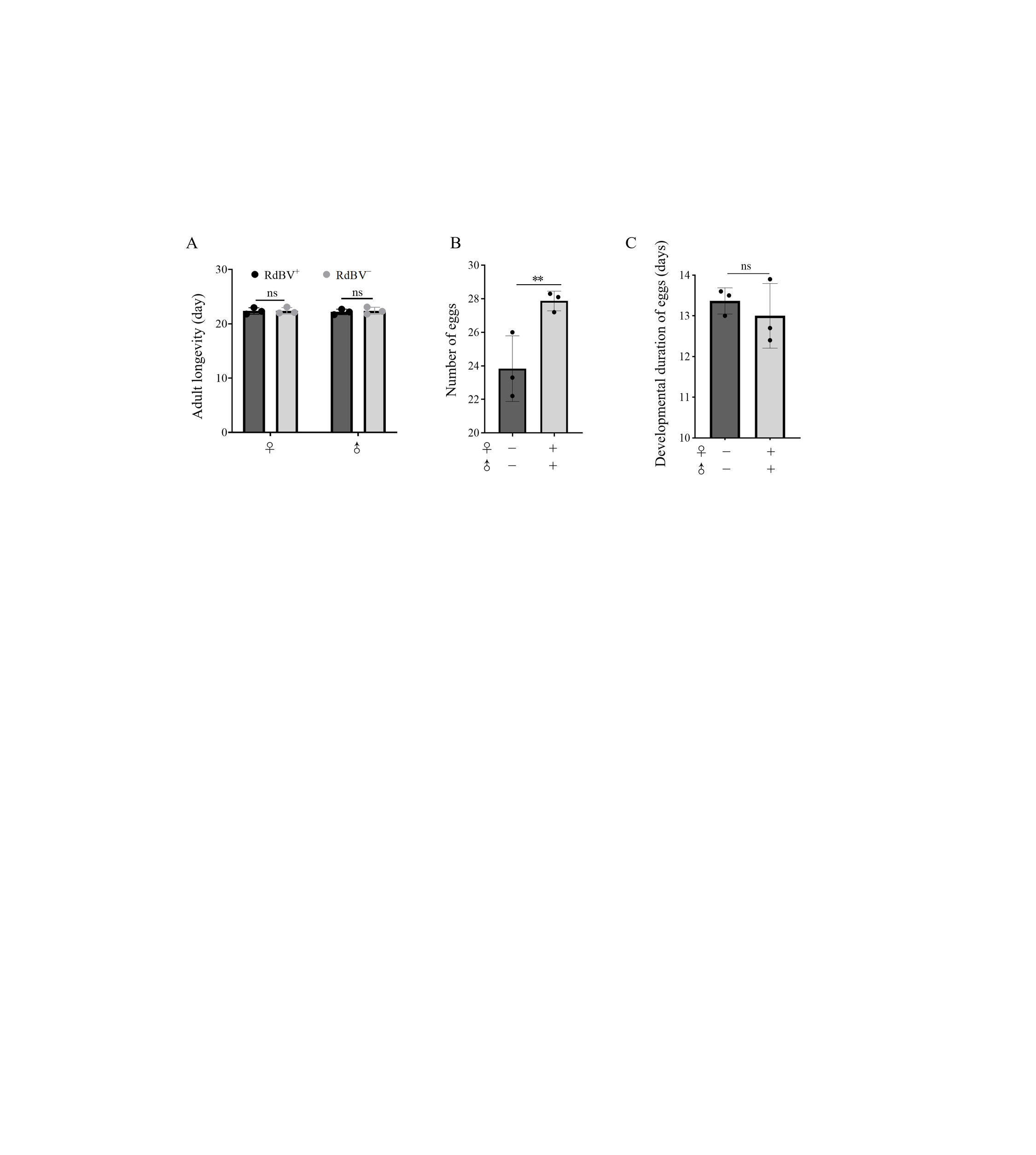

Supplement: S3 Fig — Mating combinations were established as follows: infected virgin female × infected male, and uninfected virgin female × uninfected male. (A) Effects of RdBV infection on the longevity of adults. Longevity of 50 RdBV-negative and positive female and male adults. Means (± SD) are shown from 50 insects, and represent three biological replicates (two-tailed t test). (B and C) Progeny egg number (B) and developmental duration (C) of different mating combinations. Data are presented as means (± SD) of three independent experiments of two mating combinations (two-tailed t test). *, p < 0.05; **, p < 0.01; ns, not significant. (TIF) [file ppat.1013178.s003.tif]

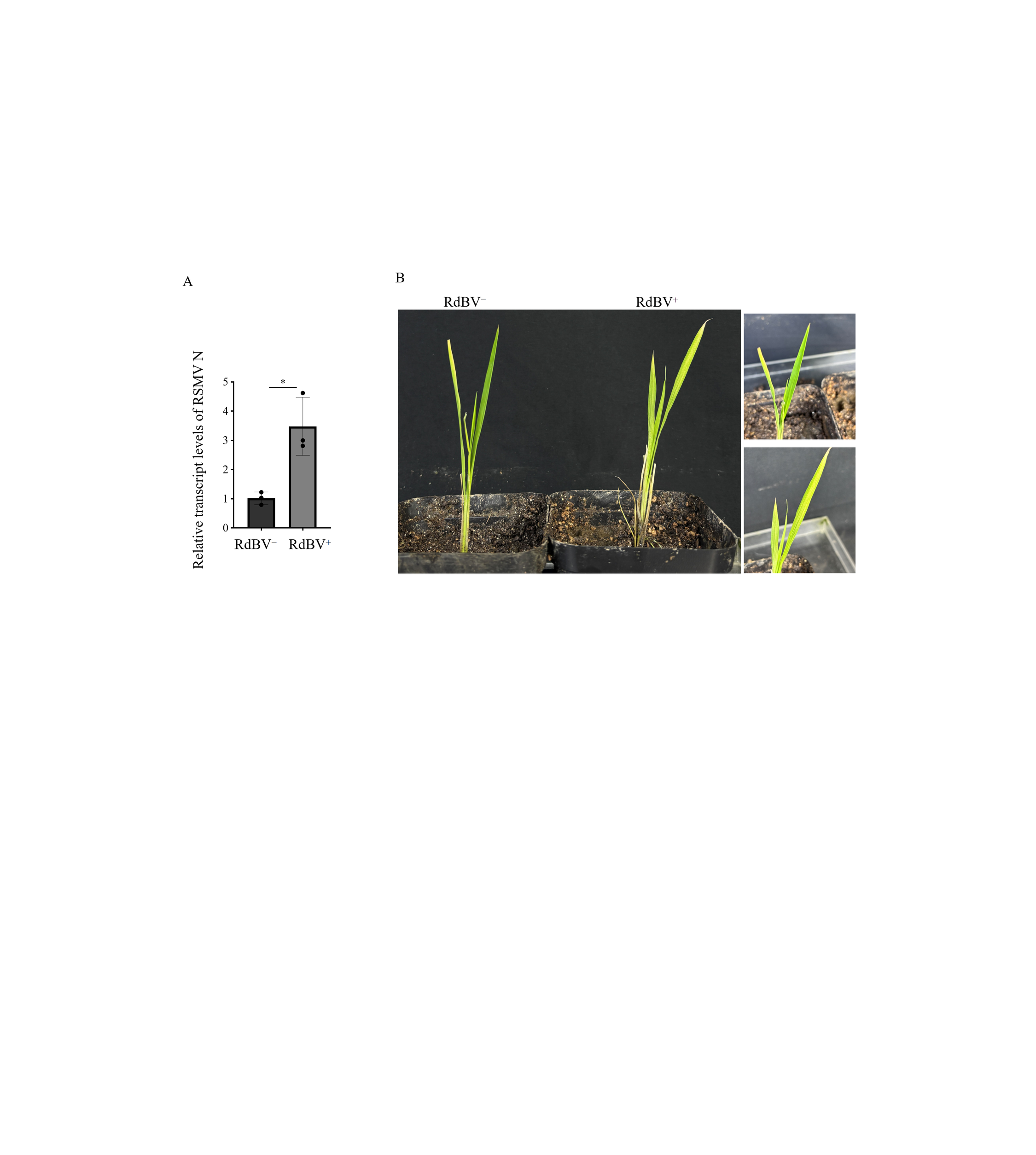

Supplement: S4 Fig — (TIF) [file ppat.1013178.s004.tif]

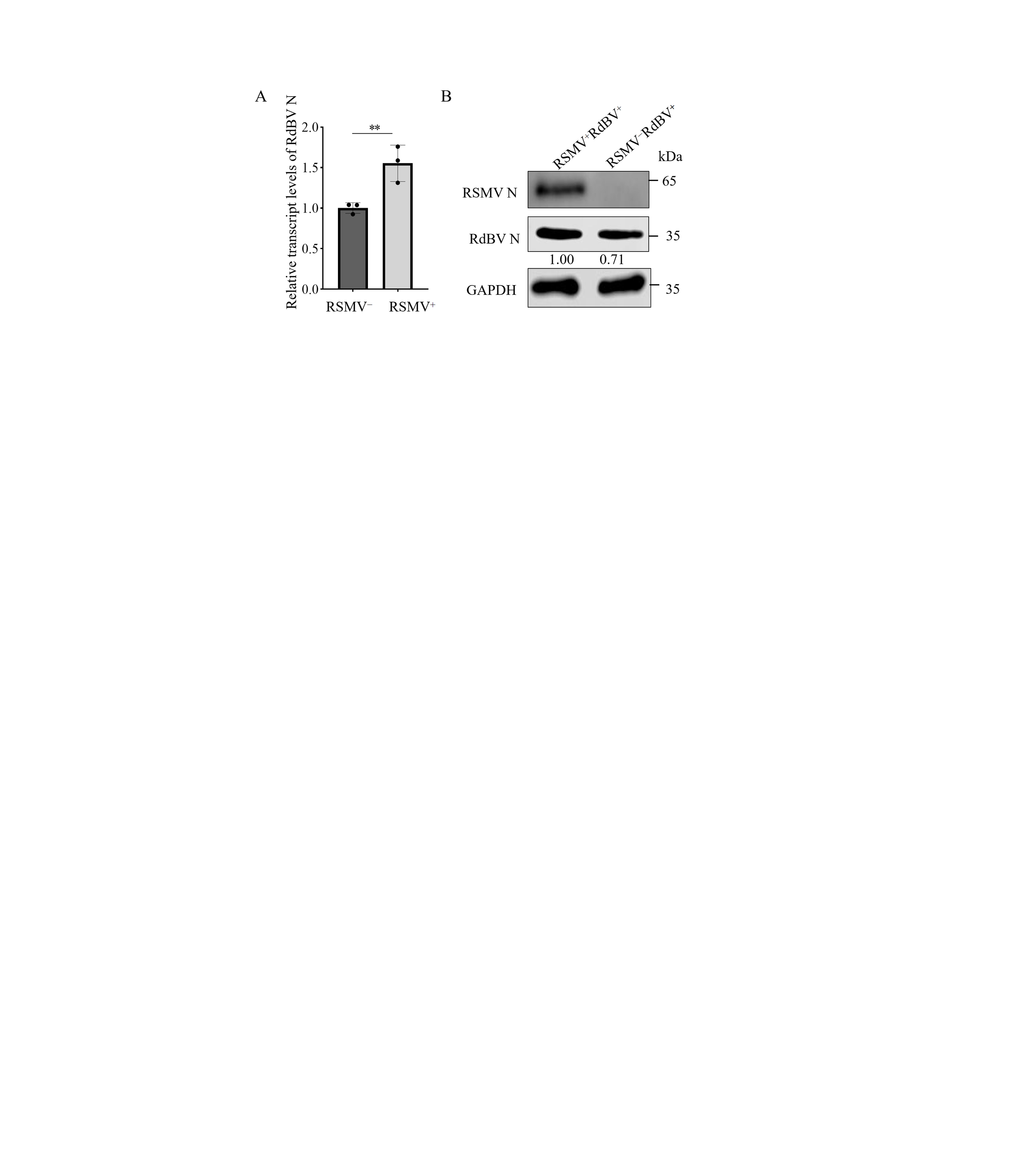

Supplement: S5 Fig — (TIF) [file ppat.1013178.s005.tif]

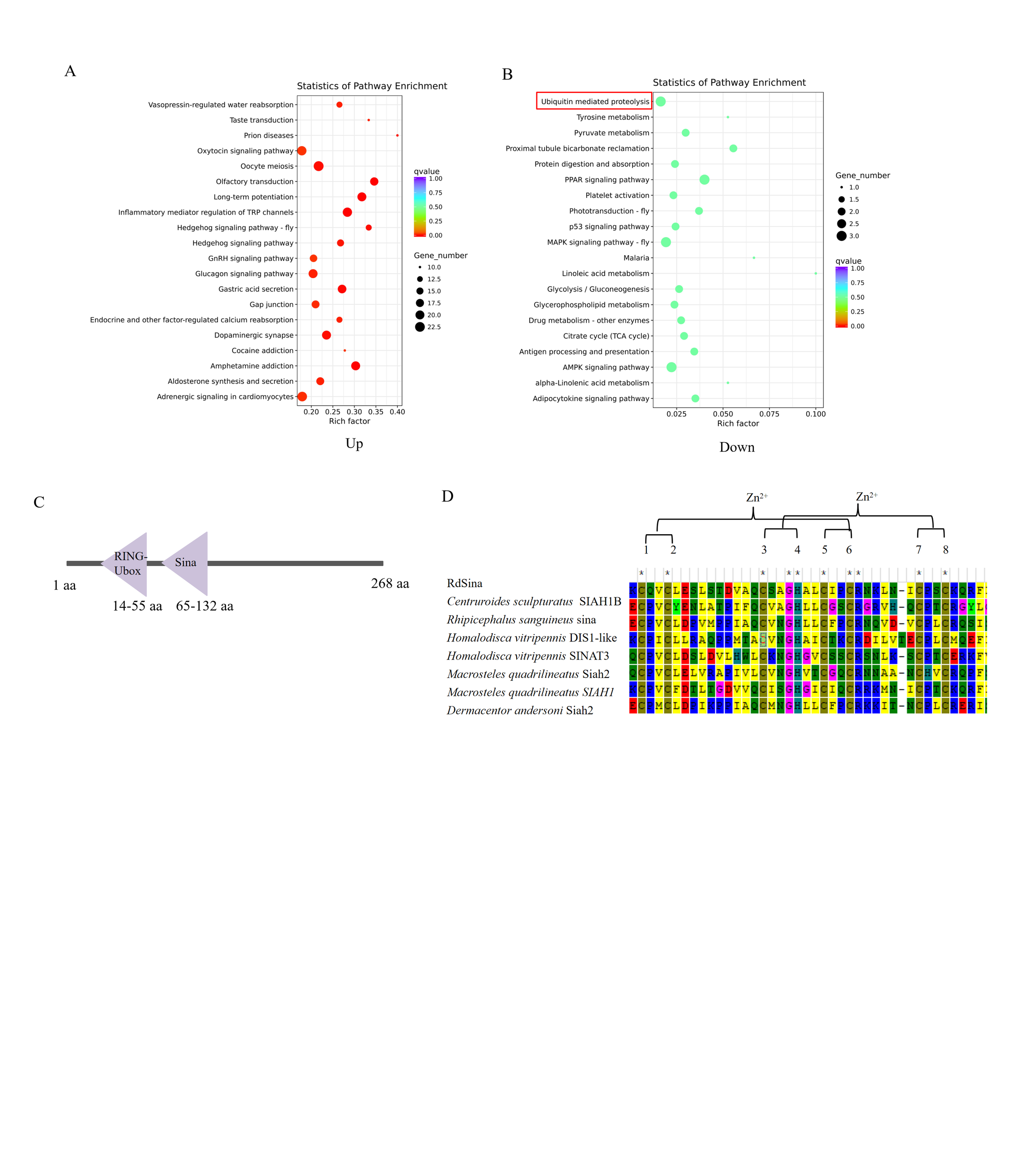

Supplement: S6 Fig — (A and B) KEGG analysis of up-regulated (A) and down-regulated genes (B) in RdBV-positive R. dorsalis compared with RdBV-negative R. dorsalis. (C) Characterization of RdSina containing a RING-Ubox domain. (D) Illustration of the RdSina C3-H-C4 RING finger composition. (TIF) [file ppat.1013178.s006.tif]

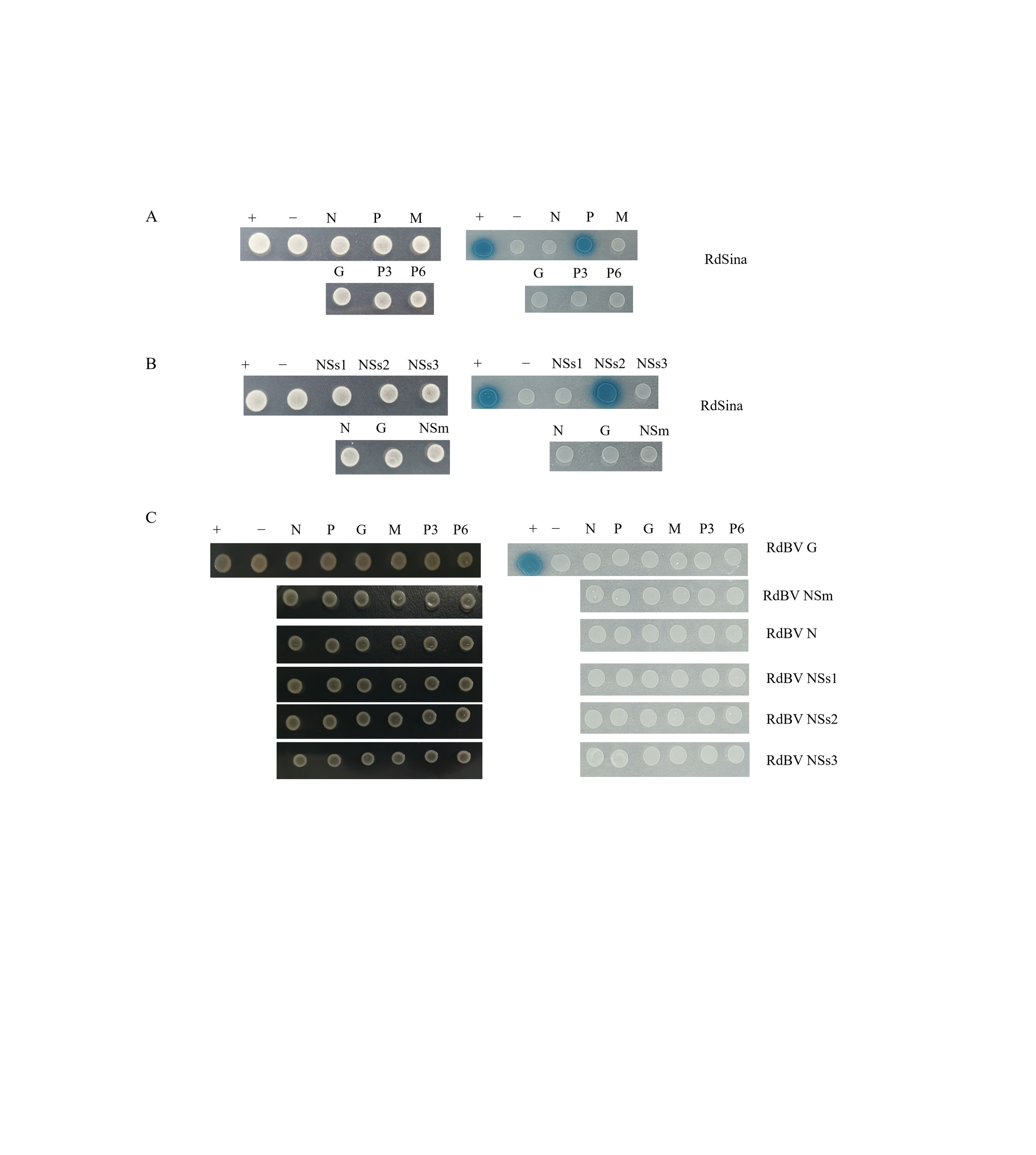

Supplement: S7 Fig — (A) Y2H assays for testing the interactions between RdSina with RdBV-encoded proteins. (B) Y2H assays for testing the interactions between RdSina with RSMV-encoded proteins. (C) Y2H assays for testing the interactions between RSMV-encoded proteins and RdBV-encoded proteins. (TIF) [file ppat.1013178.s007.tif]

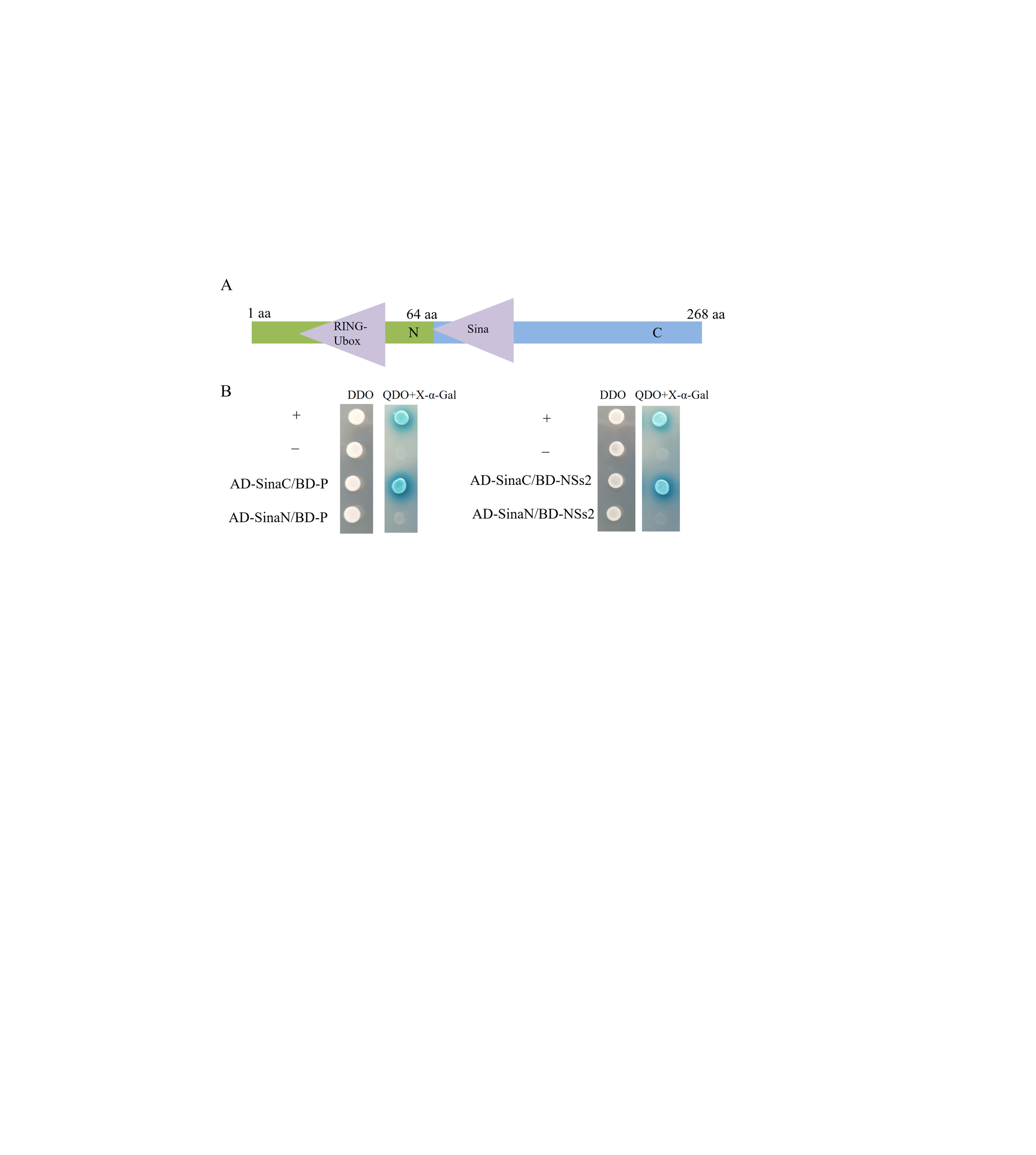

Supplement: S8 Fig — (B) Y2H assay for testing the interactions between RdSinaC (65–268 aa) and RdBV NSs2 or RSMV P. (TIF) [file ppat.1013178.s008.tif]

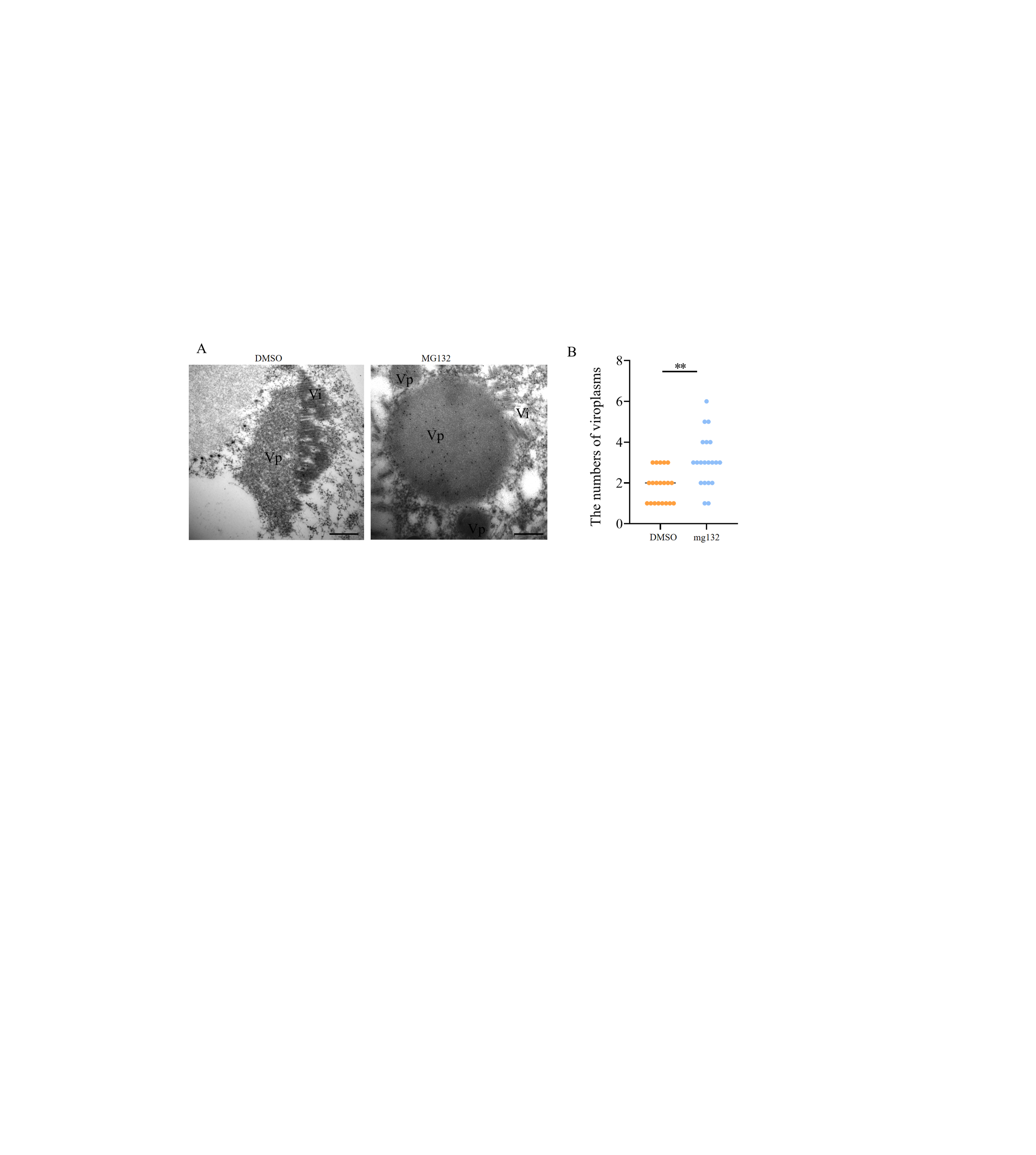

Supplement: S9 Fig — The mean number of viroplasms per midgut epithelial cell of MG132 or DMSO-treated RSMV infected R. dorsalis is shown in S9A (n = 20). Vi, virions. Vp, viroplasm. Bars: 200 nm. (TIF) [file ppat.1013178.s009.tif]

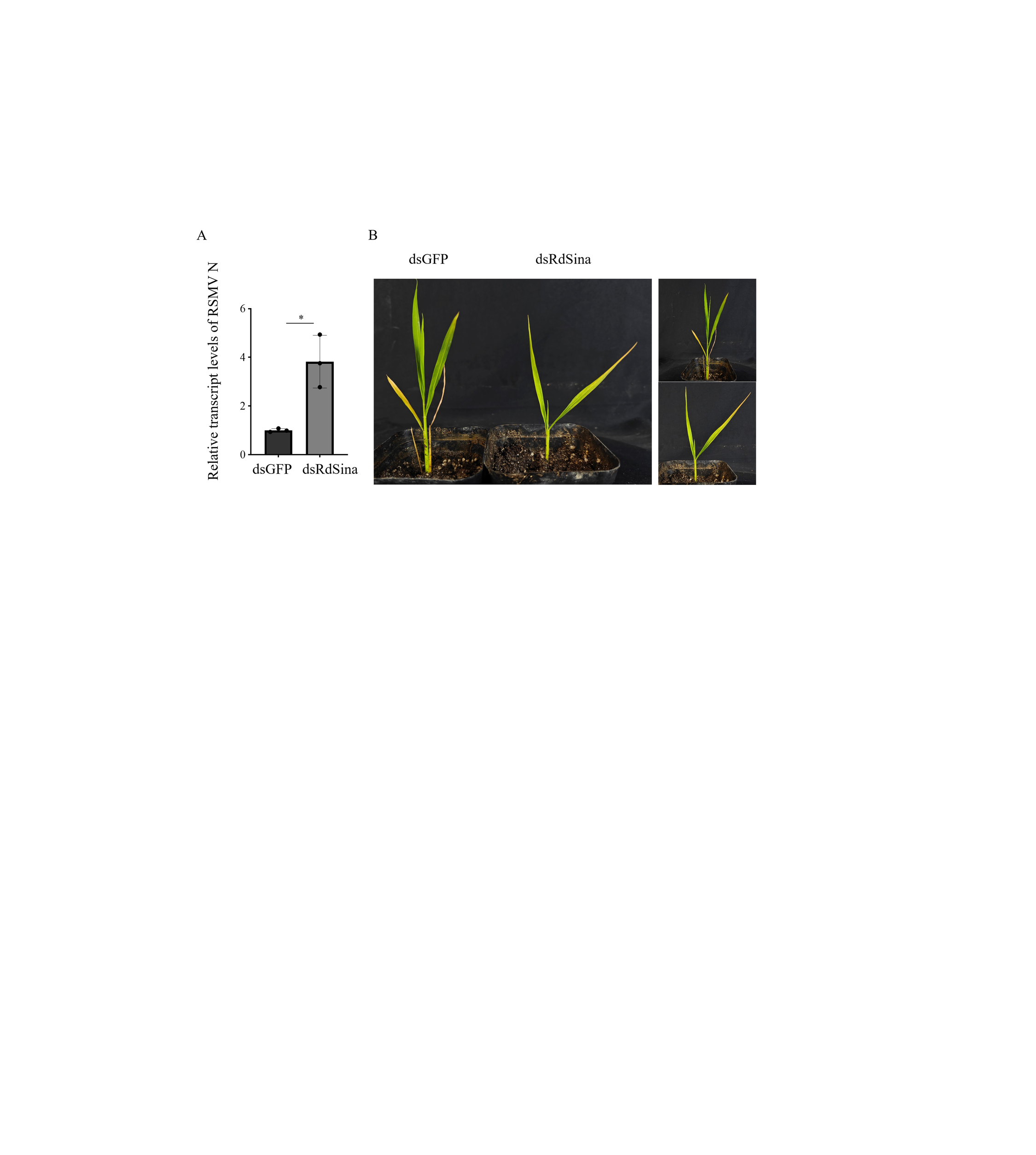

Supplement: S10 Fig — (TIF) [file ppat.1013178.s010.tif]

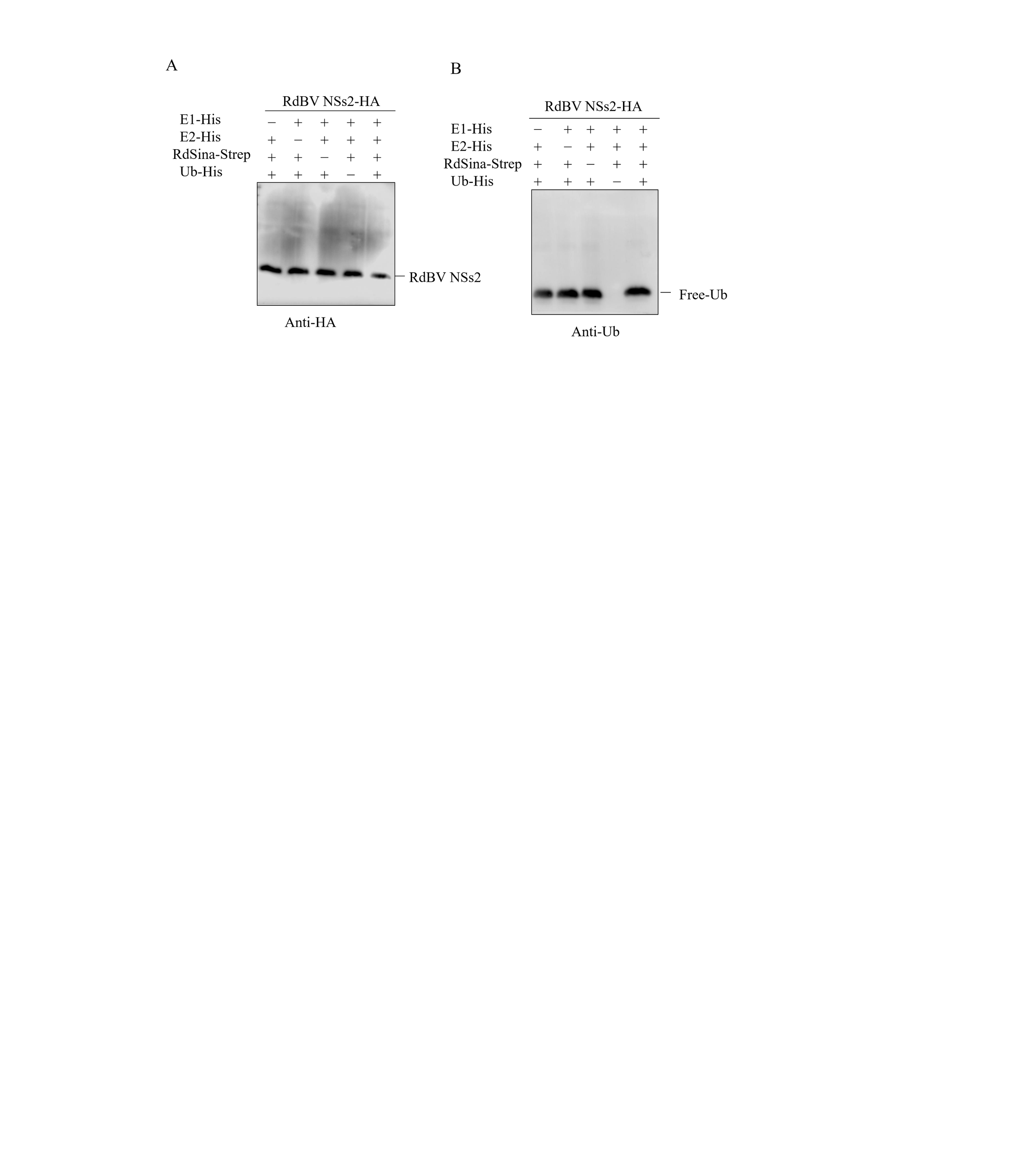

Supplement: S11 Fig — HA-NSs2 was used as a substrate for the assay. HA antibody was used in the immunoblotting assay to detect HA-NSs2 (A), and ubiquitin antibody was used to detect His-ubiquitin (B). (TIF) [file ppat.1013178.s011.tif]

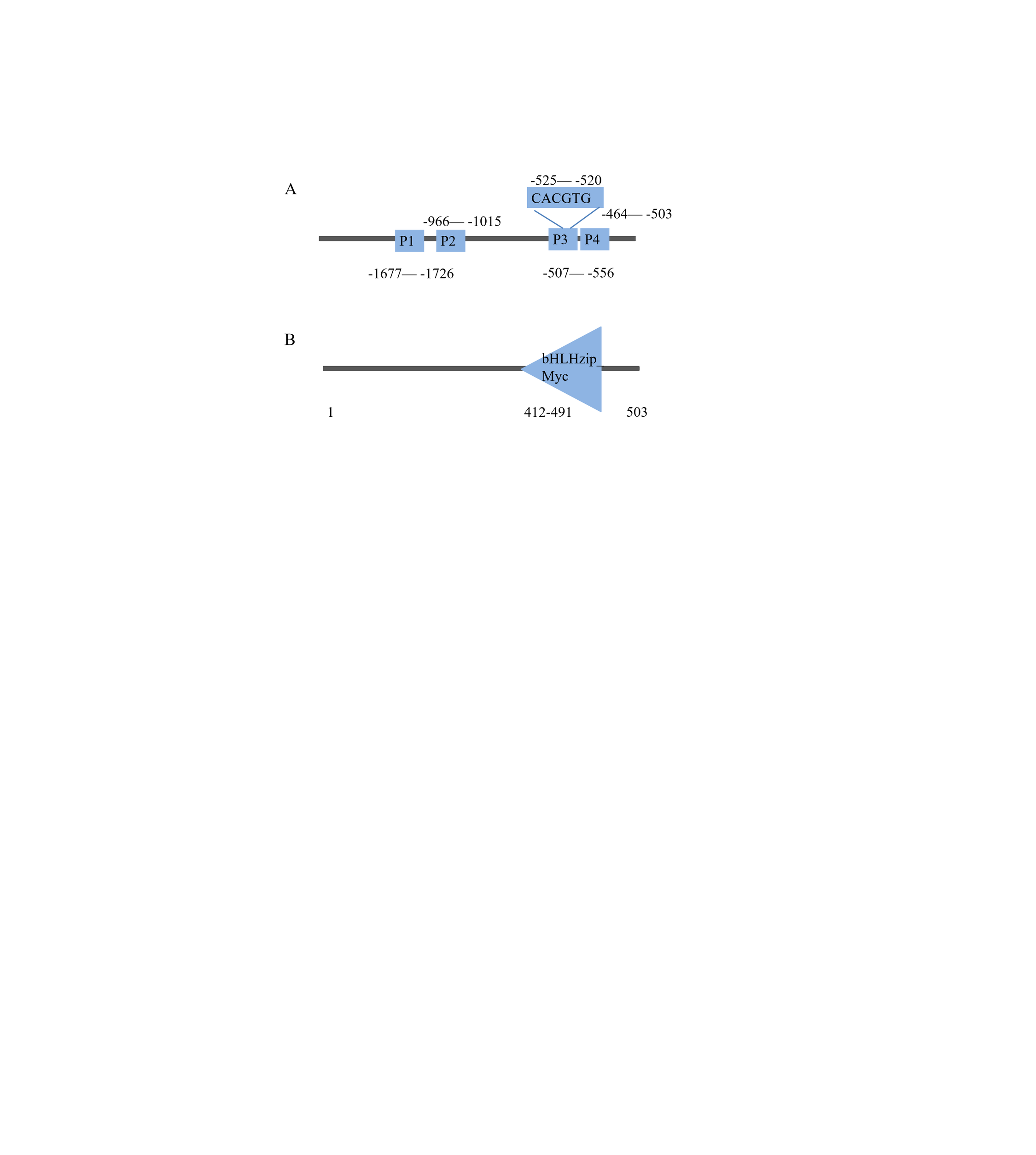

Supplement: S12 Fig — (A) Transcription factor-binding sites prediction in RdSina promoter regions. Four probe sequences (P1, P2, P3, P4) in the RdSina promoter regions were predicted to contain the transcription factor binding motif. (B) Characterization of RdMYC containing the bHLHzip_Myc domain. (TIF) [file ppat.1013178.s012.tif]

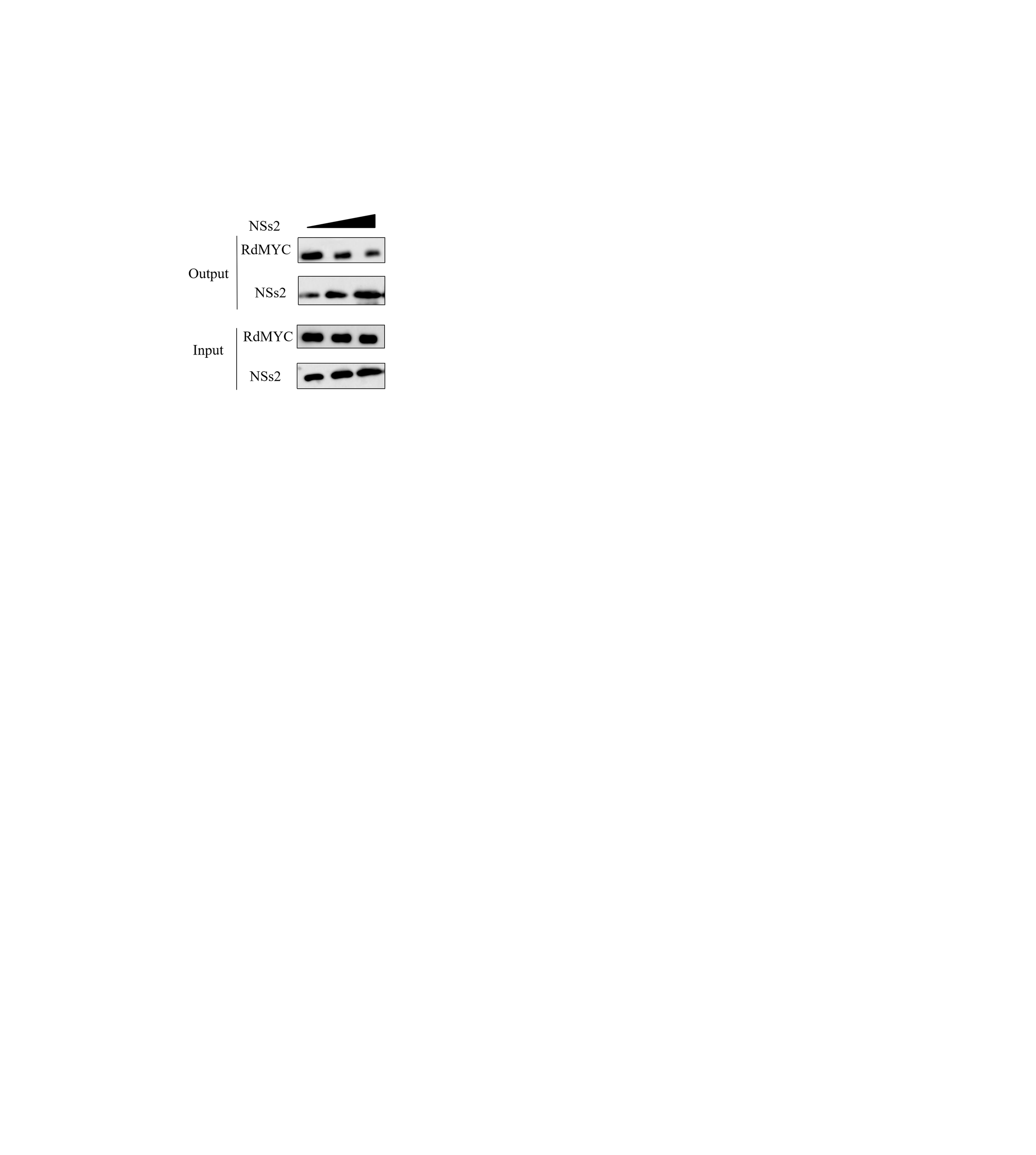

Supplement: S13 Fig — RdMYC and RdSina promoter were incubated with glutathione-Sepharose beads, then His-NSs2 was added to the beads. When the amounts of NSs2 were increased, the binding between RdMYC and RdSina promoter was decreased. (TIF) [file ppat.1013178.s013.tif]
