# Supplementary material for: An insect symbiotic virus promotes the transmission of a phytoarbovirus via inhibiting E3 ubiquitin ligase Sina
Source: PLoS Pathog. 2025 May 29;21(5):e1013178. doi: 10.1371/journal.ppat.1013178 (PMC12121772; doi:10.1371/journal.ppat.1013178)
Supplement: S1 Table — (DOCX) [file ppat.1013178.s014.docx]

**Supplementary Table 1 Percentage identity of NSs2 in comparison with other proteins.**

| Protein name | Percentage identity % |
| --- | --- |
| NP_049360.1 non-structural protein [Orthotospovirus tomatomaculae] | 21.74 |
| YP_010086065.1 nonstructural protein [Alstroemeria necrotic streak virus] | 21.74 |
| YP_009553290.1 NSs [Alstroemeria yellow spot virus] | 20.45 |
