# Supplementary material for: An insect symbiotic virus promotes the transmission of a phytoarbovirus via inhibiting E3 ubiquitin ligase Sina
Source: PLoS Pathog. 2025 May 29;21(5):e1013178. doi: 10.1371/journal.ppat.1013178 (PMC12121772; doi:10.1371/journal.ppat.1013178)
Supplement: S2 Table — (DOCX) [file ppat.1013178.s015.docx]

| **Supplementary Table 2 List of oligonucleotide primers used in this study.** | | | |
| --- | --- | --- | --- |
|  | Oligonucleotide | | Sequence (5'-3') |
|  | Detection of virus |  | |
| 1 | RdBVcheckF | TGCTAGTTGTGACTGGTCAG | |
| 2 | RdBVcheckR | GTTGGATACCCATAGTAATCC | |
| 3 | RSMVcheckF | GGAGATGAGGATTCAAAGAAGG | |
| 4 | RSMVcheckR | AATTCTTGGTCTGGAGGGATTG | |
| 5 | RdBVSF1 | AGATCAGCTAGAGTATTAGGTGC | |
| 6 | RdBVSR1 | TGAGTCAGACTCTATCAACTC | |
| 7 | RdBVMF1 | CCCAACTGCACAGACAC | |
| 8 | RdBVMR1 | CTAAAGTTAGAAGTGGGTCATG | |
| 9 | RdBVMF2 | GAATATCATGGAACTATGTAGATTG | |
| 10 | RdBVMR2 | TGCCCATCTGCCTATTTTG | |
| 11 | RdBVMF3 | CGCATCATTATCAACAATCTGG | |
| 12 | RdBVMR3 | GCAGTATGTGATATTCATATGCC | |
| 13 | RdBVMF4 | ATCACTCAGAGGTTTGGG | |
| 14 | RdBVMR4 | GGCCAGTATAGTGGGTTTAG | |
| 15 | RdBVLF1 | GTGAAGGCAAATGGTC | |
| 16 | RdBVLR1 | CAAATAAGACATTCCTCCTAGAATC | |
| 17 | RdBVLF2 | GACTAATGGTGCACAATTGG | |
| 18 | RdBVLR2 | CCTTATAATTGTGATCTTTGTACC | |
| 19 | RdBVLF3 | CGGTAACTCATCTATAGACAAAC | |
| 20 | RdBVLR3 | CACTGATGTACCTTTCATAC | |
| 21 | RdBVLF4 | GCTGTTCTGTATTCTCACTGG | |
| 22 | RdBVLR4 | GAATTATATTCACAACATTGACTTC | |
| 23 | RdBVLF5 | GTTTGTTGGGAATGGCTAC | |
| 24 | RdBVLR5 | GTCATGCTAACTATAGATGGTATAAG | |
|  |  |  | |
|  | Y2H |  | |
| 25 | AD-RdSinaN-F | CCATGGAGGCCAGTGAATTCATGGAAGTTACAGATAAGCTGCG | |
| 26 | AD-RdSinaN-R | GCTCGAGCTCGATGGATCCCTGTGGGTTTGATATTGAGGAAC | |
| 27 | AD-RdSinaC-F | CCATGGAGGCCAGTGAATTCATGTTAGTGCAAATCTTAGAAG | |
| 28 | AD-RdSinaC-R | GCTCGAGCTCGATGGATCCCTCAAGGGGTGGCTTTAACAAAC | |
| 29 | BD-RdSina-F | TGGCCATGGAGGCCGAATTCATGGAAGTTACAGATAAGCTGCG | |
| 30 | BD-RdSina-R | GCTGCAGGTCGACGGATCCCTCAAGGGGTGGCTTTAACAAAC | |
| 31 | AD-RdBV-NSs2-F | CCATGGAGGCCAGTGAATTCATGATGAGTCAGACTCTATCAACTC | |
| 32 | AD-RdBV-NSs2-R | GCTCGAGCTCGATGGATCCCTTAGCATTGAGGCATGTCTAGTAG | |
| 33 | BD-RdBV-NSs2-F | TGGCCATGGAGGCCGAATTCATGATGAGTCAGACTCTATCAACTC | |
| 34 | BD-RdBV-NSs2-R | GCTGCAGGTCGACGGATCCCTTAGCATTGAGGCATGTCTAGTAG | |
| 35 | AD-RdBV-NSs1-F | CCATGGAGGCCAGTGAATTCATGATACAGTATAGTCTTATCGTG | |
| 36 | AD-RdBV-NSs1-R | GCTCGAGCTCGATGGATCCCTCATGGTGGTGACGGTAATG | |
| 37 | BD-RdBV-NSs1-F | TGGCCATGGAGGCCGAATTCATGATACAGTATAGTCTTATCGTG | |
| 38 | BD-RdBV-NSs1-R | GCTGCAGGTCGACGGATCCCTCATGGTGGTGACGGTAATG | |
| 39 | AD-RdBV-NSs3-F | CCATGGAGGCCAGTGAATTCATGGCTCTCTTTATTGTCCTGTC | |
| 40 | AD-RdBV-NSs3-R | GCTCGAGCTCGATGGATCCCTTATAACAAGGATGACATAGTAAAGGAG | |
| 41 | BD-RdBV-NSs3-F | TGGCCATGGAGGCCGAATTCATGGCTCTCTTTATTGTCCTGTC | |
| 42 | BD-RdBV-NSs3-R | GCTGCAGGTCGACGGATCCCTTATAACAAGGATGACATAGTAAAGGAG | |
| 43 | AD-RdBV-G-F | CCATGGAGGCCAGTGAATTCATGCAGAGTTCAACCTTACTAATG | |
| 44 | AD-RdBV-G-R | GCTCGAGCTCGATGGATCCCTTACCTCACAGCATCCCTCTTG | |
| 45 | BD-RdBV-G-F | TGGCCATGGAGGCCGAATTCATGCAGAGTTCAACCTTACTAATG | |
| 46 | BD-RdBV-G-R | GCTGCAGGTCGACGGATCCCTTACCTCACAGCATCCCTCTTG | |
| 47 | BD-RdBV-NSm-F | TGGCCATGGAGGCCGAATTCATGTTTAAGTTGTTTGTGTTAGTATC | |
| 48 | BD-RdBV-NSm-R | GCTGCAGGTCGACGGATCCCTCAGATTGCATCAATTTGTCCTGG | |
| 49 | AD-RSMV-P3-F | CCATGGAGGCCAGTGAATTCATGAAGATCATCTGCAGTACTGG | |
| 50 | AD-RSMV-P3-R | GCTCGAGCTCGATGGATCCCTCAAGTAGCAAACTTGACATGG | |
| 51 | AD-RSMV-P6-F | CCATGGAGGCCAGTGAATTCATGGAGTTCAATTGGCCTTG | |
| 52 | AD-RSMV-P6-R | GCTCGAGCTCGATGGATCCCTTAGGCATCATGCAGTACCCA | |
| 53 | AD-RSMV-N-F | CCATGGAGGCCAGTGAATTCATGGCAACCGACAAGTCTTT | |
| 54 | AD-RSMV-N-R | GCTCGAGCTCGATGGATCCCTTAAGCCTTGGTCTGGAAGA | |
| 55 | BD-RSMV-P-F | TGGCCATGGAGGCCGAATTCATGAGTGTGCCAGAGGATAC | |
| 56 | BD-RSMV-P-R | GCTGCAGGTCGACGGATCCCTCACAGTGCATCATCATAGTAATCC | |
| 57 | AD-RSMV-M-F | CCATGGAGGCCAGTGAATTCATGGCCGTTCCGTGGACTGA | |
| 58 | AD-RSMV-M-R | GCTCGAGCTCGATGGATCCCCTAACTCCAGATTATACTTCCTCC | |
| 59 | BD-RSMV-G-F | TGGCCATGGAGGCCGAATTCATGATGAGGATTTCGGTCTTTC | |
| 60 | BD-RSMV-G-R | GCTGCAGGTCGACGGATCCCTTAGTCACTTTCTATAGTATATTCGG | |
|  |  |  | |
|  | expression vector in E. coil |  | |
| 61 | PGEX-NSs2-F | CGCGTGGATCCCCGAATTCCATGATGAGTCAGACTCTATCAACTC | |
| 62 | PGEX-NSs2-R | ACGATGCGGCCGCTCGAGTCTTAGCATTGAGGCATGTCTAGTAG | |
| 63 | PGEX-P-F | CGCGTGGATCCCCGAATTCCATGGCAACCGACAAGTCTTT | |
| 64 | PGEX-P-R | ACGATGCGGCCGCTCGAGTCTCACAGTGCATCATCATAGTAATCC | |
| 65 | 28b-RdBV-N-F | TGGGTCGGGATCCGAATTCGATGAATATCGACATTGCACCAGC | |
| 66 | 28b-RdBV-N-R | AGTGCGGCCGCAAGCTTGTCTCACGAAGTTGGAGATGATGAG | |
| 67 | 28b-RdSina-F | TGGGTCGGGATCCGAATTCGATGGAAGTTACAGATAAGCTGCG | |
| 68 | 28b-RdSina-R | AGTGCGGCCGCAAGCTTGTCTCAAGGGGTGGCTTTAACAAAC | |
| 69 | 28b-Ub-Strep-f | TGGGTCGGGATCCGAATTCGATGCAGATTTTTGTTAAAACGCTC | |
| 70 | 28b-Ub-Strep-r | AGTGCGGCCGCAAGCTTGTCTTTTTCGAACTGAGGGTGAGACCATCCTCCTCGGAGTCGC | |
| 71 | 28b-RSMV P-Flag-F | TGGGTCGGGATCCGAATTCGGATTACAAGGACGACGATGACAAGATGAGTGTGCCAGAGGATAC | |
| 72 | 28b-RSMV P-Flag-R | AGTGCGGCCGCAAGCTTGTCCTTGTCATCGTCGTCCTTGTAATCCAGTGCATCATCATAGTAATCC | |
| 73 | 28b-E1-F | TGGGTCGGGATCCGAATTCGATGTCTAGTGCTGAGGTGGT | |
| 74 | 28b-E1-R | AGTGCGGCCGCAAGCTTGTCTTAGGCACGAAAGGGCAAAGTG | |
| 75 | 28b-E2-F | TGGGTCGGGATCCGAATTCGATGACGTCATCGAGGCGTTT | |
| 76 | 28b-E2-R | AGTGCGGCCGCAAGCTTGTCCTACGATTTCAATCTTTTTTCAGC | |
| 77 | 28b-NSs2-HA-F | TGGGTCGGGATCCGAATTCGATGATGAGTCAGACTCTATCAACTC | |
| 78 | 28b-NSs2-HA-R | AGTGCGGCCGCAAGCTTGTCTTAAGCGTAATCTGGAACATCGTATGGGTATTAGCATTGAGGCATGTCTAGTAG | |
| 79 | 28b-RdSina-Strep-F | TGGGTCGGGATCCGAATTCGATGGAAGTTACAGATAAGCTGCG | |
| 80 | 28b-RdSina-Strep-R | AGTGCGGCCGCAAGCTTGTCTTTATTTTTCGAACTGCGGGTGGCTCCAAGGGGTGGCTTTAACAAAC | |
|  |  |  | |
|  | Sf9 cell |  | |
| 81 | Pfast-RdSina-Strep-F | CCCACCATCGGGCGCGGATCCATGGAAGTTACAGATAAGCTGCG | |
| 82 | Pfast-RdSina-Strep-R | TCGACGTAGGCCTTTGAATTCTTTTTCGAACTGAGGGTGAGACCAAGGGGTGGCTTTAACAAAC | |
| 83 | Pfast-RSMV-P-His-F | CCCACCATCGGGCGCGGATCCATGAGTGTGCCAGAGGATAC | |
| 84 | Pfast-RSMV-P-His-R | TCGACGTAGGCCTTTGAATTCttaATGGTGATGGTGATGATGCAGTGCATCATCATAGTAATCC | |
| 85 | pfast-NSs2-HA-F | CCCACCATCGGGCGCGGATCCATGATGAGTCAGACTCTATCAACTC | |
| 86 | pfast-NSs2-HA-R | TCGACGTAGGCCTTTGAATTCTTAAGCGTAATCTGGAACATCGTATGGGTATTAGCATTGAGGCATGTCTAGTAG | |
|  |  |  | |
|  | RT-qPCR |  | |
| 87 | RSMV N-F | AGTCTTGGCAAGCCATCTGT | |
| 88 | RSMV N-R | GCGGCCTTTTTAGTATCCGC | |
| 89 | RSMV P-F | CCACTGAGGACCAAGTCAGG | |
| 90 | RSMV P-R | TCTTAGGAAGACCGGAGCGT | |
| 91 | RdMYC-F | GGAAACTCAAGGCGACCCAC | |
| 92 | RdMYC-R | CTTAGCTCGCAACAGTAACTCG | |
| 93 | RdSina-F | GAGACATGTACCTTTGGGGC | |
| 94 | RdSina-R | GTGAGGGCTCAAGTCAATGC | |
| 95 | RdBV N-F | GGCTGGCACTTAGGAGACTT | |
| 96 | RdBV N-R | GTCGACTGTGCTGCTTGTTG | |
| 97 | EF1-F | CAGTGAGAGCCGTTTTGAG | |
| 98 | EF1-R | AGGGCATCTTGTCAGAGGGC | |
|  |  |  | |
|  | RNAi |  | |
| 99 | dsGFP-F | GGATCCTAATACGACTCACTATAGGATGGCTAGCAAAGGAGAAG | |
| 100 | dsGFP-R | GGATCCTAATACGACTCACTATAGGTAAGAGAAAGTAGTGACAAG | |
| 101 | dsRdMYC-F | attctctagaagcttaatacgactcactatagggATGCCGATCTGCAAGTTCGGCG | |
| 102 | dsRdMYC-R | attctctagaagcttaatacgactcactatagggTGAGCCTCCAGATGTCGGTCTGGG | |
| 103 | dsRdSina-F | ATTCTCTAGAAGCTTAATACGACTCACTATAGGGATGGAAGTTACAGATAAGCTGCG | |
| 104 | dsRdSina-R | ATTCTCTAGAAGCTTAATACGACTCACTATAGGGGGCGTGTTCTATCTTTCATGC | |
|  |  |  | |
|  | EMSA |  | |
| 105 | P1 | TTCATCGTTATATATAAATACATAAATAAATTAATTAATTATGATGGGAT | |
| 106 | P2 | AATTCTGGTTATATATATTGGGTAAGATATTAACACCTACAGAGTCAGCG | |
| 107 | P3 | TTTGGCGCCCTCTATCTTGTGCGCCCCCGGGCACGTGCCCAGTGTGCCCA | |
| 108 | P4 | ATAGAAACGGGCCTGGGTTACAACCCCCAAACCCCCCCCC | |
| 109 | P3mutant | TTTGGCGCCCTCTATCTTGTGCGCCCCCGGGCAAAAACCCAGTGTGCCCA | |
|  |  |  | |
|  | Dual-luciferase reporter assay |  | |
| 110 | pIB-RdMYC-F | CGAATTTAAAGCTTGGTACCATGCCGATCTGCAAGTTCG | |
| 111 | pIB-RdMYC-R | CGAACCGCGGGCCCTCTAGATCAGTATCGGTGTTGGTGTTG | |
| 112 | pIB-NSs2-F | CGAATTTAAAGCTTGGTACCATGATGAGTCAGACTCTATCAACTC | |
| 113 | pIB-NSs2-R | CGAACCGCGGGCCCTCTAGATTAGCATTGAGGCATGTCTAGTAG | |
| 114 | pGL3-SinaP-F | CGTGCTAGCCCGGGCTCGAGTAGTAAGCGAGAAAGTTTTTT | |
| 115 | pGL3-SinaP-R | AGTACCGGAATGCCAAGCTTTGTAACAAACAAGTTATTATTAG | |
